# Longitudinal Trajectories of Stress and Positive Aspects of Dementia Caregiving: Findings From the IDEAL Programme

**DOI:** 10.1093/geronb/gbae097

**Published:** 2024-05-30

**Authors:** Catherine Quinn, Laura D Gamble, Robin G Morris, Claire Pentecost, Jennifer M Rusted, Linda Clare

**Affiliations:** Centre for Applied Dementia Studies, University of Bradford, Bradford, UK; Wolfson Centre for Applied Health Research, Bradford, UK; Population Health Sciences Institute, Newcastle University, Newcastle Upon Tyne, UK; Department of Psychology, King’s College London, Institute of Psychiatry, Psychology and Neuroscience, London, UK; University of Exeter Medical School, Exeter, UK; School of Psychology, University of Sussex, Brighton, UK; University of Exeter Medical School, Exeter, UK; NIHR Applied Research Collaboration South-West Peninsula, Exeter, UK; (Psychological Sciences Section)

**Keywords:** Alzheimer’s disease, Carer, Cohort, Well-being

## Abstract

**Objectives:**

Understanding what influences changes over time in caregiver well-being is important for the development of effective support. This study explores differences in trajectories of caregiver stress and positive aspects of caregiving (PAC).

**Methods:**

Caregivers of community-dwelling individuals with mild-to-moderate dementia at baseline from the IDEAL cohort were interviewed at baseline (*n* = 1,203), 12 months (*n* = 917), and 24 months (*n* = 699). Growth mixture models identified multiple growth trajectories of caregiver stress and PAC in the caregiver population. Associations between study measures and trajectory classes were examined using multinomial logistic regression and mixed-effects models.

**Results:**

Mean stress scores increased over time. A 4-class solution was identified: a “high” stable class (8.3%) with high levels of stress, a “middle” class (46.1%) with slightly increasing levels of stress, a “low” class (39.5%) with initial low levels of stress which slightly increased over time, and a small “increasing” class (6.1%) where stress level started low but increased at a steeper rate. Mean PAC scores remained stable over time. A 5-class solution was identified: 3 stable classes (“high,” 15.2%; “middle,” 67.6%; “low” 9.3%), a small “increasing” (3.4%) class, and 1 “decreasing” class (4.5%). For stable classes, positive ratings on study measures tended to be associated with lower stress or higher PAC trajectories and vice versa. Those with “increasing” stress also had worsening trajectories of several study measures including depression, relationship quality, competence, and ability to cope.

**Discussion:**

The findings highlight the importance of identifying caregivers at risk of increased stress and declining PAC and offering them targeted support.

Informal caregivers are family members or friends who provide support to someone with dementia. The need for better help for caregivers of people with dementia has been identified in policy and practice guidelines (Alzheimer’s Association, 2023; Gauthier et al., 2022). These caregivers provide extensive support for people with dementia. Caregiving can have a significant negative impact on caregivers’ health and well-being (Alzheimer’s Association, 2023). Understanding how caregiver well-being changes over time and the factors that might influence this is important for the development of effective help. Typically, caregiver well-being has been explored by focusing on specific domains, most commonly the negative aspects of well-being characterized in terms of levels of burden or stress. However, caregiving can also be a positive experience (Kramer, 1997; Quinn & Toms, 2019) and research has shown that positive and negative aspects of caregiving can have independent associations with caregiver well-being (Quinn et al., 2019). While there is a growing evidence base about what might influence caregivers’ experiences of stress or positive aspects of caregiving (PAC), less is known about whether experiences change over time and what factors might influence trajectories of change. A better understanding of this would help in the development of more holistic methods of supporting caregivers.

Theoretically, the negative aspects of caregiving tend to be viewed in terms of what causes stress or burden, or alternatively the impact on caregiving outcomes (e.g., Pearlin et al., 1990). Models of stress (e.g., Folkman, 1997) indicate that stress can be mitigated through effective coping, but caregiving stressors can multiply or change over time, stretching coping resources to the extent that they are not fully effective. Because studies of caregiving have typically focused on burden, rather than stress, the review of the evidence base here will predominantly draw on studies exploring this aspect. A few studies have explored how burden changes over time; reviews by Chiao et al. (2015) identified 3 studies and van den Kieboom et al. (2020) identified 11 studies. Out of these, only one study focused on stress. Despite wide differences in follow-up periods, they did indicate that, overall, both burden (Brodaty et al., 2014; Conde-Sala et al., 2014; Froelich et al., 2009; Jones et al., 2017; Reed et al., 2020) and stress (Svendsboe et al., 2018) increase over time. However, Kajiwara et al. (2018) reported no difference in burden scores over 12 months and Aguera-Ortiz et al. (2010) reported a small decrease in burden at 12-month follow-up. A few studies have explored predictors of burden over time; these include transitions (e.g., Bleijlevens et al., 2015), behavioral symptoms and functional limitations in the person with dementia (e.g., Brodaty et al., 2014; Jones et al., 2017; Mohamed et al., 2010; Reed et al., 2020), caregiver personality, and sense of competence (e.g., Reis et al., 1994; van der Lee et al., 2017). A review by van der Lee et al. (2014), which included cross-sectional studies, identified seven core domains that influence burden, encompassing the behavior, mood, cognitive function and self-care ability in the person with dementia, and caregiver support, physical and psychological health, competence, coping, and personality. There is little research into what might influence different trajectories of burden over time and only one study has focused on caregiver stress. Studies have identified differences in trajectories of burden due to caregiver ethnicity (Skarupski et al., 2009) and kin relationship (Vinas-Diez et al., 2017). Bangerter et al. (2019) examined the influence of resources and transitional events on role captivity and role overload over 12 months. The study Svendsboe et al. (2018), which is the only study to explore caregiving stress, only examined the influence of diagnosis (dementia with Lewy bodies [DLB] or Alzheimer’s disease [AD]) and living situation (living at home or nursing home) on stress over a 3-year period. To our knowledge, only one study has explored the influence of multiple factors on trajectories of burden in caregivers of community-dwelling people with AD (Conde-Sala et al., 2014). This study identified one group demonstrating stable scores, one group with moderate baseline burden that increased in severity, and a third group with high baseline burden that decreased in severity. There were some differences in the individuals within these groups, providing initial evidence that longitudinally there could be differences in burden. As far as we are aware, no study has explored the influence of multiple factors on trajectories of caregiver stress.

There is growing evidence that caregivers have positive experiences in providing care (Yu et al., 2018). Specifically, they describe benefits for themselves such as personal accomplishment and gratification, benefits for the person with dementia, and dyadic benefits relating to the continuation of the relationship (Quinn et al., 2024; Yu et al., 2018). Identifying positive experiences in providing care can have a beneficial impact on caregiver well-being (e.g., Quinn & Toms, 2019). Yet there is conceptual unclarity about the role that positive experiences have for caregivers. In the Stress Process Model (Pearlin et al., 1990), gain is conceptualized as a mediator of the caregiving process. Feelings of gain are linked to the enhancement of the self and inner growth, whereas barriers to the development of this result in “secondary intrapsychic strains.” In other theoretical perspectives, positive experiences may act as a means to adapt to stressors or form a type of coping (Folkman, 1997; Fredrickson, 2004; Tennen & Affleck, 2002). Theoretically then, positive experiences might buffer the effects of caregiving stress. Although there is a suggestion that positive and negative dimensions of caregiving exist at opposite ends of a continuum, it is also possible that both states can co-occur at the same time (Quinn et al., 2019). There are limitations in the predominantly cross-sectional evidence base, and there is little evidence about whether and how positive experiences change over time. A systematic review (Quinn & Toms, 2019) identified only a few studies that had incorporated measures of positive experiences in longitudinal studies. Although three studies measured positive experiences longitudinally, they did not report the data for each time point (Cohen et al., 1994; Lethin et al., 2017; Reis et al., 1994). Lévesque et al. (1998) collected data at baseline and 1 year later and found a nonsignificant decrease in positive experiences at follow-up. Gold et al. (1995) conducted assessments at baseline and 6 months later and, although not reporting a significant change over time, reported that gender (female), social support, and health were significant predictors of positive experiences.

In conclusion, a review of longitudinal studies provides little evidence concerning how stress changes over time, although evidence from research on burden indicates there might be different trajectories. Additionally, few studies have explored changes in PAC over time and none have looked at differences within trajectories. Understanding more about potential caregiving stress and PAC changes over time, and what factors may influence this, would help in the development of more effective support for caregivers. The aims of this study were as follows:

1. To explore changes in caregiver stress and PAC over time.
2. To identify whether there are groups with different trajectories of caregiver stress and PAC over time and if so, to identify factors associated with these trajectories. Specifically, we will examine experiences of caregiving (stress, social restriction, PAC, competence, and coping), psychological characteristics and health (neuroticism, self-esteem, and depression), quality of the relationship with the person with dementia, physical health, and abilities of the person with dementia (functional activities, dependence, presence and severity of distress, and cognitive function).

We hypothesized that both caregiver stress and PAC would change over time and it would be possible to identify groups with different trajectories on these measures.

## Method

### Design

The study analyzes longitudinal data from the “Improving the Experience of Dementia and Enhancing Active Life (IDEAL) study” (Clare et al., 2014). Participants were identified from 29 National Health Service sites within Great Britain. Data were collected using questionnaire packs that caregivers self-completed. This study used data collected at three assessment time points at 12-month intervals. The IDEAL study was approved by the Wales Research Ethics Committee 5 (reference 13/WA/0405), and the Ethics Committee of the School of Psychology, Bangor University (reference 2014-11684), and is registered with UKCRN (#16593).

### Participants

The IDEAL cohort was formed by recruiting community-dwelling individuals diagnosed with mild-to-moderate dementia of any type, with a Mini-Mental State Examination (MMSE; Folstein et al., 1975) score ≥15 on enrollment, and able to provide informed consent. Where the person with dementia was willing to take part, the caregiver was approached to participate. For the purposes of this paper, we used information that caregivers provided about their own experiences and those of the person with dementia. People with dementia were also administered a measure of cognitive functioning. At baseline (Time 1, T1), there were 1,277 caregivers. We excluded caregivers of people who moved into residential care during the study period and any caregivers who were substituted for the originally participating caregiver at T2 or T3 resulting in 1,203 caregivers at T1, 917 at T2, and 699 at T3.

### Measures

The IDEAL study assessments included an extensive set of measures (for details, see Clare et al., 2014). This study used a specific subset of these measures, with measure selection guided by previous findings and the domains identified by van den Kieboom et al. (2020). See Supplementary Material for a more detailed description of the measures included in the analyses.

### Demographic Measures

Measures included caregiver status (kin relationship), sex, age, living situation, education, socioeconomic status, and hours spent per day caregiving. For the care recipient, information was collected on the diagnosis.

### Study Measures

#### Measures about the caregiver

Caregivers completed measures relating to experiences of caregiving. Caregiver stress was measured with the 15-item Relative Stress Scale (example item: Do you ever feel frustrated with your relative/friend?), and the 2-item Modified Social Restriction Scale explored how easy it is to find someone to look after the care recipient. The nine-item PAC scale explored positive experiences (example item: Providing help to my relative/friend has made me feel appreciated) whilst the three-item Caregiving Competence Scale measured perceived competency (example item: How often do you feel that you are doing a good job as a caregiver?). A single item (Do you think you cope well as a caregiver?) measured coping.

Caregivers also completed measures pertaining to psychological characteristics and health. These were the mini-IPIP neuroticism measure (example item: I seldom feel blue), the 10-item Rosenberg Self-Esteem Scale (example item: I feel that I’m a person of worth, at least on an equal plane with others), and the 20-item Center for Epidemiologic Studies—Depression Scale-Revised (CES-D-R; example item: I felt sad). The relationship with the person with dementia was measured using the five-item Positive Affect Index, which measured current relationship quality with the care recipient (example item: How often do you and your relative friend do things together). Physical health was measured using a single item of self-rated health (Overall, how would you rate your health in the past 4 weeks?).

#### Measures about the care recipient

Caregivers completed the Functional Activities Questionnaire (example item: Can your relative/friend shop alone for clothes, household necessities, and groceries?) and the Dependence scale (example item: Does your relative/friend need to be tube fed?) about the abilities of the care recipient. The presence and severity of, and distress due to, neuropsychiatric symptoms were assessed using the Neuropsychiatric Inventory (NPI) Questionnaire (example item: Is your relative/friend stubborn and resistive to help from others?). The cognitive ability of the care recipient was assessed using the MMSE.

### Statistical Analysis

Version 7 of the IDEAL data set was used. Using Mplus v8.2, we employed growth mixture modeling (GMM) to identify multiple classes of growth trajectories of stress and PAC. More details on model selection can be found in Supplementary Material and Supplementary Figures 1 and 2. Based on model fit indices, class size, and theoretical interpretations, a four-class solution was taken forward for stress and a five-class solution for PAC. For stress, the average latent class probabilities ranged from 0.81to 0.83 and the entropy was 0.68 (Supplementary Tables 1 and 2) and for PAC, the average latent class probabilities ranged from 0.75 to 0.90 and the entropy was 0.79 (Supplementary Tables 3 and 4). The posterior probability of class membership was used to investigate associations of baseline measures with the intercept (baseline) and slope (change per timepoint) of each class through multinomial regression. Adjusted odds ratios are reported alongside 95% confidence intervals. The adjusted odds ratios are the relative odds of being in a given class over the reference class, given exposure to the variable of interest, and after controlling for other covariates in the model.

Mixed-effects modeling examined associations of class membership with trajectories of scores on measures assessed longitudinally. Mixed-effects modeling was conducted in R using the *lme4* package, with random effects to account for interindividual variation. For continuous measures, residuals were checked for normality. For all continuous measures, a gamma distribution with a log link was fitted. A binomial distribution with a log link was fitted for coping, and a Poisson model was fitted for the count of the number of NPI symptoms. All models were adjusted for caregiver sex, caregiver age, diagnosis type, and caregiver status.

Missing data on outcome measures were handled using full-information maximum-likelihood estimation with the assumption that data are missing at random. Missing data on covariates were imputed using multiple imputation with chained equations in Mplus, generating 25 data sets. Estimates were combined according to Rubin’s rules.

To further explore the relationship between stress and PAC, a parallel process model was specified to jointly model both stress and PAC in the same growth mixture model. Based on model fit indices, the three-class solution (GMM-CI) was taken forward. Class solutions beyond three resulted in classes corresponding to the three classes of the three-class solution, but with increasing numbers of very small classes (<2%; Supplementary Table 5). The average latent class probabilities ranged from 0.80 to 0.96 and the entropy was high at 0.891 (Supplementary Table 6).

## Results

Our study cohort comprised 1,203 caregivers at T1, 917 at T2, and 699 at T3. A comparison of caregivers who remained and did not remain in the study is presented in Supplementary Table 7. Caregiver and care-recipient characteristics and scores on study variables are summarized in Supplementary Table 8. At baseline, caregiver mean age was 69 years, two thirds were females caring for a man, and more than 80% of caregivers were spouses or partners. Overall, mean stress increased over time and PAC remained stable.

### Stress

**Figure 1.**
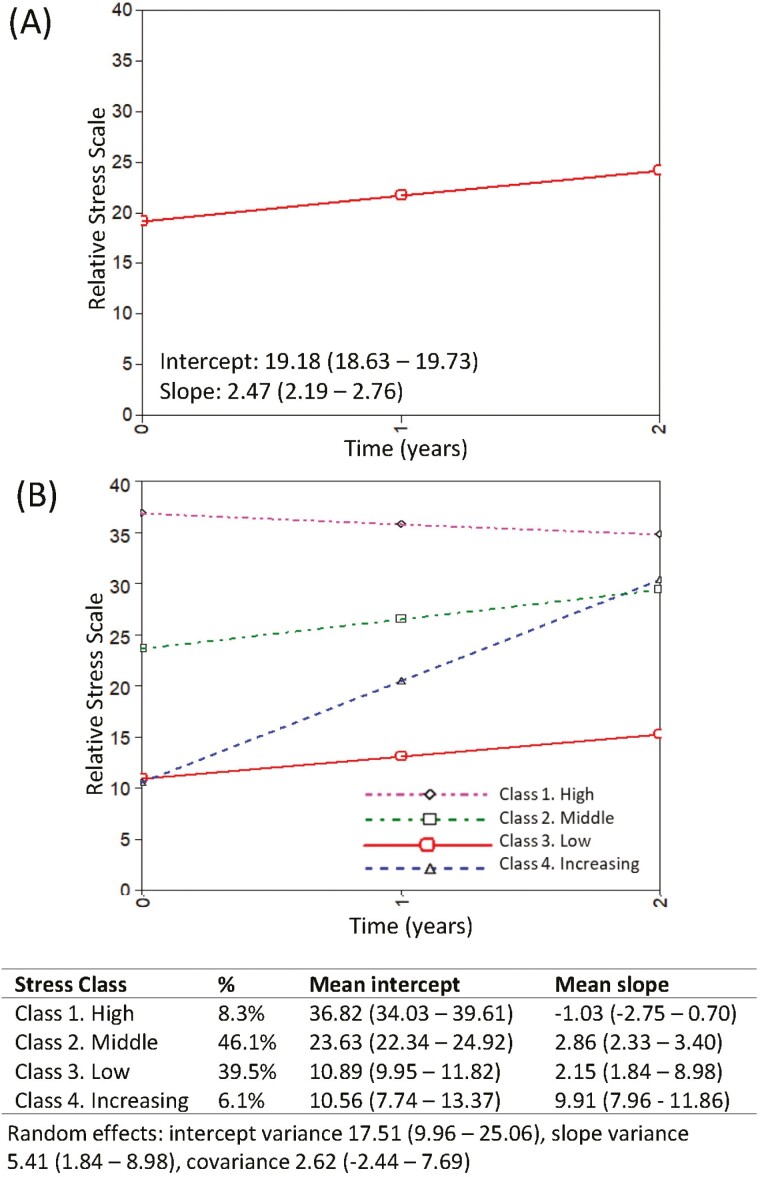
Trajectories of caregiving stress over time. Panel (**A**) The mean intercept and slope of the Relative Stress Scale for caregivers. Panel (**B**) Trajectories of stress determined from the GMM-CI model; Class 1: High, Class 2: Middle, Class 3: Low, and Class 4: Increasing. The mean intercepts and slopes associated with each class are shown, as are the intercept and slope variances which are equal across classes. 95% confidence intervals are displayed in brackets. GMM-CI = growth mixture model-class invariant

Scores for stress were available for 1,181 caregivers at one or more time points (1,120 at T1, 857 at T2, and 657 at T3). As shown in Figure 1A, mean stress scores increased at each time point (2.47, 95% 2.19–2.76). We investigated heterogeneity in trajectories of stress using growth mixture models and based on model fit, class size, and theoretical interpretations, a four-class solution was taken forward. The resulting classes were a stable class with high levels of stress (Class 1, hereafter referred to as “High,” 8.3%), a middle class with slightly increasing levels of stress (Class 2, hereafter referred to as “Middle,” 46.1%), a class with low levels of stress, again with a slight increase over the study period (Class 3, hereafter referred to as “Low,” 39.5%), and a small class which started low but increased at a steeper rate (Class 4, hereafter referred to as “Increasing,” 6.1%). Trajectories, alongside fixed and random effects, are shown in Figure 1B, and individuals within each class are plotted in Supplementary Figure 3. Characteristics of the caregivers in each class are shown in Supplementary Table 9. Given some uncertainty in class membership, further analyses took into account the probabilities of each individual being a member of each class. Associations of baseline measures with class membership were examined using multinomial regression (Table 1).

**Table 1.** Associations of Baseline Measures With Classes of Stress

Baseline measures	C1 High (8.3%) (ref: C2 Middle) OR (95% CI)	C3 Low (39.5%) (ref: C2 Middle) OR (95% CI)	C4 Increasing (6.1%) (ref: C2 Middle) OR (95% CI)	C4 Increasing (6.1%) (ref: C1 Low) OR (95% CI)
Caregiver age	1.01 (0.97–1.05)	1.00 (0.99–1.02)	1.02 (0.98–1.06)	1.02 (0.98–1.05)
Caregiver sex (male)	0.35 (0.10–1.27)	2.29 (1.53–3.43)*	0.79 (0.21–3.02)	0.35 (0.09–1.36)
Diagnosis				
AD	Ref	Ref	Ref	Ref
VaD	1.42 (0.49–4.12)	1.36 (0.73–2.53)	0.43 (0.05–3.61)	0.31 (0.04–2.83)
Mixed AD/VaD	1.67 (0.77–3.65)	1.02 (0.64–1.64)	1.35 (0.53–3.40)	1.31 (0.48–3.61)
FTD	1.54 (0.35–6.76)	0.74 (0.34–1.61)	NE	NE
PDD/DLB	1.14 (0.36–3.63)	0.43 (0.19–0.94)*	NE	NE
Other	1.69 (0.29–9.75)	0.14 (0.02–1.11)	NE	NE
Caregiver status (family/friend)	1.18 (0.38–3.66)	2.53 (1.35–4.73)*	0.62 (0.14–2.75)	0.24 (0.05–1.18)
Measures about the care recipient				
FAQ-I	1.09 (1.03–1.15)*	0.88 (0.85–0.90)*	0.85 (0.79–0.92)*	0.97 (0.89–1.05)
Dependence-I	1.36 (1.14–1.63)*	0.61 (0.54–0.68)*	0.62 (0.48–0.79)*	1.01 (0.77–1.33)
NPI distress	1.29 (1.15–1.46)*	0.72 (0.67–0.78)*	0.54 (0.38–0.76)*	0.75 (0.53–1.06)
NPI severity	1.32 (1.18–1.48)*	0.72 (0.66–0.78)*	0.58 (0.42–0.80)*	0.80 (0.58–1.12)
NPI symptoms	1.64 (1.27–2.13)*	0.60 (0.54–0.68)*	0.44 (0.31–0.62)*	0.72 (0.51–1.03)
MMSE	0.99 (0.91–1.09)	1.09 (1.04–1.15)*	1.10 (0.96–1.26)	1.01 (0.87–1.16)
Measures about the caregiver				
MSRS	1.55 (1.22–1.96)*	0.55 (0.46–0.66)*	0.98 (0.64–1.50)	1.77 (1.08–2.93)*
PAC	0.93 (0.89–0.98)*	1.04 (1.02–1.07)*	1.03 (0.93–1.14)	0.99 (0.89–1.11)
Competence	0.61 (0.45–0.81)*	1.70 (1.46–1.98)*	2.09 (1.40–3.12)*	1.23 (0.81–1.87)
Neuroticism (mini-IPIP)	1.58 (1.31–1.90)*	0.72 (0.67–0.78)*	0.70 (0.59–0.84)*	0.97 (0.80–1.18)
Self-esteem (RSE)	0.85 (0.78–0.93)*	1.22 (1.15–1.29)*	1.25 (1.10–1.42)*	1.02 (0.90–1.17)
CES-D-R	1.17 (1.11–1.23)*	0.77 (0.71–0.82)*	0.76 (0.67–0.85)*	0.99 (0.86–1.14)
PAI current	0.79 (0.70–0.90)*	1.28 (1.21–1.36)*	1.44 (1.23–1.68)*	1.12 (0.96–1.32)
Self-rated health	0.74 (0.54–1.02)	1.82 (1.50–2.19)*	1.13 (0.75–1.69)	0.62 (0.40–0.97)*
Coping (never/sometimes vs often/always)	4.11 (2.04–8.31)*	0.23 (0.13–0.41)*	0.36 (0.10–1.32)	1.55 (0.35–6.93)

*Notes*: AD = Alzheimer’s disease; aOR = adjusted odds ratio; CES-D-R = Center for Epidemiologic Studies—Depression Scale-Revised; CI = confidence interval; DLB = dementia with Lewy bodies; FAQ-I = functional activities questionnaire–informant-rated; FTD = frontotemporal dementia; MMSE = Mini-Mental State Examination; MSRS = modified social restriction scale; NE = not estimated; NPI = neuropsychiatric inventory; PAC = positive aspects of caregiving; PAI = positive affect index; PDD = Parkinson’s disease dementia; Ref = reference; VaD = vascular dementia. Models were adjusted for caregiver sex, caregiver age, diagnosis type, caregiver status.

^*^Confidence intervals do not span one.

The biggest differences were seen between the High, Middle, and Low classes of stress, distinguished by their scores at baseline since they are relatively stable over time. Compared with the Middle class, those in the High class were at baseline more likely to score higher on neuroticism, report more NPI symptoms, and report increased severity of symptoms and increased distress at symptoms. They were more likely to report lower self-esteem, competence, relationship quality, PAC, higher social restriction and depression, and less ability to cope. The person they provided care for was also more likely to be functionally impaired and more dependent at baseline. Generally, the opposite was seen for the Low stress group when compared to the Middle group. At baseline, they scored lower on neuroticism, reported fewer NPI symptoms, lower severity of symptoms, and less distress at symptoms. They were likely to report better self-esteem, competence, relationship quality, PAC, and coping and had fewer social restrictions and less depression. The care recipient was more likely to have better cognition (MMSE) and be less functionally impaired and less dependent. Additionally, they were more likely to report better self-rated health, were more likely to be male, to be a family/friend caregiver, and less likely to care for someone with Parkinson’s disease dementia (PDD) or DLB.

The Increasing stress class had similar baseline scores of stress to the Low stress class, and they had similar baseline scores to the Low stress class on most study measures. We, therefore, compared the Increasing class with the Low class to see if we could identify any factors associated with Increasing stress when scores at baseline were similar. Those in the Increasing class were likely to be more socially restricted and have poorer self-rated health at baseline, but actual numerical differences were quite small. No other differences were found; therefore, we investigated further by exploring whether increasing stress may be explained by changes in study measures over time using mixed-effects models (Table 2 and Supplementary Table 10). Those in the Increasing stress class were more likely to report increasing numbers and severity of NPI symptoms and increasing distress at symptoms compared to those in the Low class. They were more likely to show a greater decline in competence, relationship quality, PAC, and self-rated health, and become less able to cope. There was a greater likelihood of increasing depression and social restriction and a greater reported decline in the care recipient’s functional abilities and dependence compared to the Low class.

**Table 2.** Associations of Classes of Stress and Study Measures Over Time

Measures	C1 High (8.3%) (ref: C2 Middle)	C3 Low (39.5%) (ref: C2 Middle)	C4 Increasing (6.1%) (ref: C2 Middle)	C4 Increasing (6.1%) (ref: C1 Low)
Measures about the care recipient				
FAQ-I	0.87 (0.78–1.02)	1.06 (0.99–1.14)	1.68 (1.44–1.97)*	1.58 (1.37–1.82)*
Dependence-I	0.92 (0.82–1.03)	1.00 (0.94–1.06)	1.23 (1.09–1.40)*	1.23 (1.08–1.47)*
NPI distress	0.69 (0.55–0.87)*	0.87 (0.77–0.98)*	2.12 (1.65–2.74)*	2.40 (1.85–3.12)*
NPI severity	0.73 (0.60–0.89)*	0.91 (0.82–1.00)	1.83 (1.47–2.28)*	2.02 (1.60–2.54)*
NPI symptoms	0.86 (0.76–0.96)*	0.96 (0.89–1.04)	1.69 (1.43–1.99)*	1.75 (1.47–2.09)*
Measures about the caregiver				
MSRS	0.97 (0.89–1.06)	0.96 (0.91–1.00)	1.09 (0.98–1.20)	1.14 (1.01–1.28)*
PAC	1.01 (0.95–1.08)	1.03 (1.00–1.07)*	0.98 (0.91–1.05)	0.95 (0.91–0.98)*
Competence	1.08 (1.03–1.14)*	1.01 (0.98–1.04)	0.94 (0.89–1.00)*	0.94 (0.88–0.99)*
CES-D-R	0.85 (0.68–1.06)	0.84 (0.75–0.94)*	1.44 (1.14–1.83)*	1.75 (1.37–2.24)*
PAI current	1.00 (0.95–1.05)	1.03 (1.00–1.05)*	0.92 (0.87–0.98)*	0.90 (0.85–0.96)*
Self-rated health	0.95 (0.87–1.04)	1.04 (0.99–1.09)	0.93 (0.84–1.03)	0.90 (0.81–1.00)
Coping (binomial)	0.64 (0.28–1.44)*	0.52 (0.29–0.94)*	1.64 (0.72–3.77)	2.81 (1.14–6.94)*

*Notes*: CES-D-R = Center for Epidemiologic Studies—Depression Scale-Revised; FAQ-I = functional activities questionnaire—informant-rated; MSRS = modified social restriction scale; NPI = neuropsychiatric inventory; PAC = positive aspects of caregiving; PAI = positive affect index; ref = reference. Data values represent rate ratios (95% confidence interval) for all measures, except for coping, for which the values represent the adjusted odds ratio (95% confidence interval). Models were adjusted for caregiver sex, caregiver age, diagnosis type, and caregiver status.

^*^Confidence intervals do not span one.

### Positive Aspects of Caregiving

**Figure 2.**
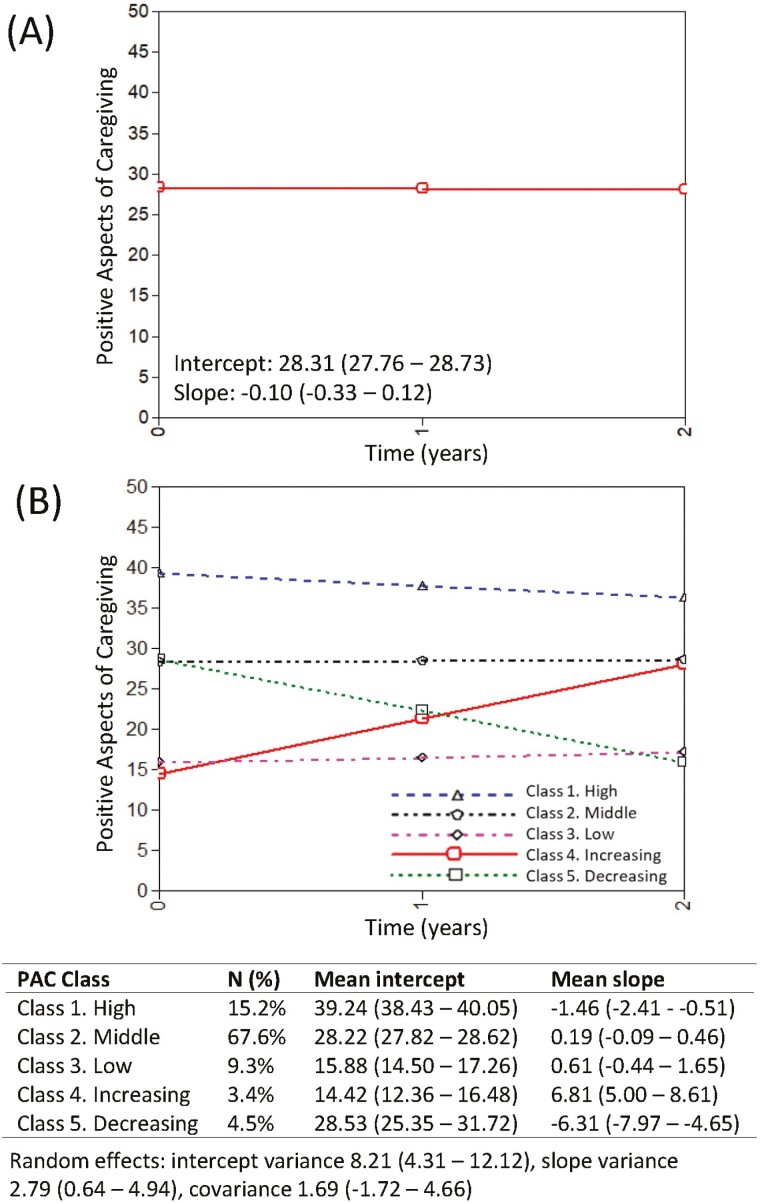
Trajectories of positive aspects of caregiving over time. Panel (**A**) The mean intercept and slope of positive aspects of caregiving (PAC). Panel (**B**) Trajectories of PAC determined from the GMM-CI model; Class 1: High, Class 2: Middle, Class 3: Low, Class 4: Increasing, and 5: Decreasing. The mean intercepts and slopes associated with each class are shown, as are the intercept and slope variances which are equal across classes. 95% confidence intervals are displayed in brackets. GMM-CI = growth mixture model-class invariant

Scores for PAC were available for 1,191 caregivers at one or more time points (1,155 at T1, 862 at T2, and 663 at T3). As shown in Figure 2A, mean PAC scores remained stable over time (−0.10, 95% −0.33 to 0.12). Again, we investigated heterogeneity in trajectories of PAC using growth mixture models. Based on model fit indices, class size, and theoretical interpretations, a five-class solution was taken forward. Most people remained stable over time resulting in three classes with little change: a class with high PAC (Class 1, hereafter referred to as “High,” 15.2%), a large class with middle range scores on PAC (Class 2, hereafter referred to as “Middle,” 67.6%), and a class with low levels of PAC (Class 3, hereafter referred to as “Low,” 9.3%). Two small classes with changing PAC scores were identified; a class with increasing PAC scores (Class 4, hereafter referred to as “Increasing,” 3.4%) and a class with decreasing PAC scores (Class 5, hereafter referred to as “Decreasing,” 4.5%). Trajectories, alongside fixed and random effects, are shown in Figure 2B, and individuals within each class are plotted in Supplementary Figure 4. Characteristics of the caregivers in each class are shown in Supplementary Table 11. Given some uncertainty in class membership, further analyses considered the probabilities of each individual being a member of each class and explored associations of baseline measures with class membership using multinomial regression (Table 3).

**Table 3.** Classes of Positive Aspects of Caregiving (PAC) and Associations of Baseline Measures With Class

Baseline measures	C1 High (15.2%) (ref: C2 Middle) OR (95% CI)	C3 Low (9.3%) (ref: C2 Middle) OR (95% CI)	C4 Increasing (3.4%) (ref: C2 Middle) OR (95% CI)	C4 Increasing (3.4%) (ref: C3 Low) OR (95% CI)	C5 Decreasing (4.5%) (ref: C2 Middle) OR (95% CI)
Age	1.00 (0.98–1.03)	1.01 (0.99–1.03)	1.00 (0.98–1.03)	1.07 (0.98–1.17)	1.03 (0.98–1.08)
Sex (male)	1.93 (1.25–2.99)*	0.52 (0.24–1.15)	0.58 (0.13–2.59)	0.62 (0.06–6.85)	0.78 (0.23–2.71)
Diagnosis AD VaD Mixed AD/VaD FTD PDD/DLB Other	Ref 0.85 (0.42–1.74) 0.97 (0.57–1.65) 1.57 (0.59–4.22) 1.04 (0.45–2.43) 0.67 (0.13–3.37)	Ref 0.50 (0.15–1.65) 1.00 (0.48–2.06) 2.78 (0.88–8.77) 1.05 (0.36–3.02) 0.84 (0.15–4.80)	Ref 0.43 (0.06–3.27) 0.47 (0.10–2.32) 0.70 (0.02–23.83) 0.27 (0.01–11.47) NE	Ref 0.62 (0.01–55.7) 0.97 (0.21–4.49) 0.82 (0.02–42.98) 0.47 (0.01–26.90) NE	Ref NE 1.28 (0.35–4.61) 3.78 (0.51–28.13) 2.05 (0.40–10.47) NE
Caregiver status (family/friend)	1.44 (0.76–2.72)	NE	2.70 (0.27–26.71)	NE	1.02 (0.09–11.85)
Measures about the care recipient					
FAQ-I	1.00 (0.97–1.02)	1.01 (0.97–1.06)	1.01 (0.92–1.11)	1.00 (0.88–1.13)	0.99 (0.93–1.05)
Dependence-I	0.95 (0.87–1.05)	0.92 (0.80–1.05)	1.07 (0.77–1.49)	1.17 (0.77–1.77)	0.91 (0.77–1.08)
NPI distress	0.96 (0.92–1.01)	1.06 (0.99–1.14)	0.90 (0.63–1.29)	0.85 (0.56–1.28)	0.96 (0.88–1.05)
NPI severity	0.96 (0.91–1.02)	1.08 (1.02–1.14)*	0.76 (0.54–1.07)	0.70 (0.49–0.99)*	0.93 (0.81–1.07)
NPI symptoms	0.93 (0.85–1.03)	1.16 (0.89–1.52)	0.67 (0.18–2.50)	0.58 (0.12–2.73)	0.84 (0.66–1.09)
MMSE	0.99 (0.93–1.04)	1.02 (0.96–1.10)	0.93 (0.79–1.10)	0.91 (0.75–1.10)	1.13 (0.97–1.31)
Measures about the caregiver					
RSS	0.95 (0.92–0.97)*	1.08 (1.04–1.13)*	0.97 (0.78–1.20)	0.89 (0.71–1.12)	1.02 (0.96–1.08)
MSRS	0.72 (0.60–0.87)*	1.65 (1.12–2.43)*	0.82 (0.34–1.96)	0.50 (0.15–1.61)	0.84 (0.50–1.42)
Competence	1.71 (1.46–2.00)*	0.60 (0.46–0.79)*	1.31 (0.74–2.33)	2.17 (1.04–4.55)*	0.97 (0.69–1.37)
Neuroticism (mini-IPIP)	0.95 (0.89–1.01)	0.98 (0.87–1.11)	1.08 (0.82–1.41)	1.09 (0.78–1.54)	0.88 (0.75–1.04)
Self-esteem (RSE)	1.10 (1.05–1.16)*	0.95 (0.87–1.04)	1.29 (1.11–1.49)*	1.36 (1.13–1.63)*	0.93 (0.81–1.06)
CES-D-R	0.95 (0.91–1.00)	1.05 (1.01–1.09)*	0.94 (0.81–1.10)	0.90 (0.75–1.08)	1.03 (0.96–1.10)
PAI current	1.16 (1.09–1.23)*	0.82 (0.76–0.89)*	1.22 (1.04–1.43)*	1.49 (1.24–1.78)*	0.96 (0.85–1.08)
Self-rated health	1.00 (0.83–1.20)	1.05 (0.75–1.46)	0.97 (0.49–1.92)	0.93 (0.38–2.28)	0.76 (0.35–1.67)
Coping (never/sometimes vs often/always)	0.16 (0.06–0.47)*	2.64 (1.35–5.17)*	2.69 (0.72–10.09)	1.02 (0.18–5.67)	2.54 (0.70–9.23)

*Notes:* AD = Alzheimer’s disease; CES-D-R = Center for Epidemiologic Studies—Depression Scale-Revised; CI = confidence intervals; DLB = dementia with Lewy bodies; FAQ-I = functional activities questionnaire—informant-rated; IPIP = International Personality Item Pool; MMSE = mini-mental state examination; MSRS = modified social restriction scale; NE = not estimated; NPI = neuropsychiatric inventory; OR = adjusted odds ratio; PAI = positive affect index; PDD = Parkinson’s disease dementia; Ref = reference category; RSE = Rosenberg Self-Esteem Scale; RSS = relative stress scale; TD = frontotemporal dementia; VaD = vascular dementia. Models are adjusted for caregiver sex, caregiver age, diagnosis type, and caregiver status.

^*^Confidence intervals do not span one.

In contrast to the findings about stress, there were relatively few measures that differed between classes for PAC. Again, the Middle class was the reference category for the comparison of the three stable classes. At baseline, those with Low PAC were more likely to have severe NPI symptoms, lower competence, and poorer relationship quality, and were coping less well. They were more likely to be depressed, socially restricted, and stressed. Those in the high PAC class were likely to have higher self-esteem, competence, and relationship quality, to cope better, and to be less socially restricted and stressed. They were also more likely to be male. Those in the Increasing PAC class had similar baseline levels of PAC to the Low class but were more likely to have better self-esteem and relationship quality at baseline, as well as less severe NPI symptoms and higher competence. There were no differences between the Decreasing class and the Middle class (both had similar baseline values) that could explain the reasons for the decline. We explored whether the decline might be explained by changes in study measures over time using mixed-effects models (Table 4 and Supplementary Table 12). Those in the Decreasing class, compared to the Middle class, were more likely to have a steeper decline in competence, relationship quality, and become less able to cope. They were also more likely to become more depressed and to rate the care recipient as being more functionally impaired.

**Table 4.** Associations of PAC Classes With Measures Across Time Points

Baseline measures	C1 High (15.2%) (ref: C2 Middle)	C3 Low (9.3%) (ref: C2 Middle)	C4 Increasing (3.4%) (ref: C2 Middle)	C4 Increasing (3.4%) (ref: C3 Low)	C5 Decreasing (4.5%) (ref: C2 Middle)
Measures about the care recipient					
FAQ-I	1.01 (0.92–1.11)	1.03 (0.92–1.16)	1.03 (0.85–1.25)	0.93 (0.81–1.22)	1.23 (1.00–1.51)*
Dependence-I	1.03 (0.96–1.11)	1.06 (0.97–1.16)	0.98 (0.84–1.14)	0.92 (0.76–1.12)	0.99 (0.84–1.16)
NPI distress	0.97 (0.84–1.12)	1.13 (0.94–1.76)	1.11 (0.83–1.48)	0.98 (0.66–1.47)	1.29 (0.95–1.76)
NPI severity	1.01 (0.88–1.16)	1.17 (0.98–1.39)	1.15 (0.87–1.53)	1.02 (0.72–1.45)	1.20 (0.89–1.60)
NPI symptoms	1.00 (0.91–1.10)	1.04 (0.94–1.15)	1.08 (0.89–1.29)	1.04 (0.83–1.30)	1.04 (0.85–1.26)
MMSE	1.02 (0.96 -1.09)	0.99 (0.92–1.08)	0.91 (0.79–1.04)	0.93 (0.79–1.11)	0.90 (0.77–1.04)
Measures about the caregiver					
RSS	1.08 (1.01–1.17)*	1.02 (0.93–1.12)	0.98 (0.84–1.14)	0.96 (0.77–1.19)	1.10 (0.94–1.29)
MSRS	1.00 (0.93–1.07)	1.03 (0.95–1.12)	0.96 (0.83–1.10)	1.00 (0.80–1.13)	1.05 (0.91–1.22)
Competence	0.99 (0.96–1.02)	0.98 (0.94–1.03)	1.03 (0.96–1.10)	1.05 (0.96–1.14)	0.89 (0.83–0.96)*
CES-D-R	1.05 (0.91–1.21)	1.11 (0.92–1.33)	0.94 (0.69–1.27)	0.87 (0.59–1.26)	1.40 (1.03–1.92)*
PAI current	1.00 (0.97–1.03)	0.98 (0.94–1.02)	1.02 (0.95–1.08)	1.04 (0.96–1.13)	0.92 (0.86–0.98)*
Self-rated health	1.02 (0.96–1.08)	1.03 (0.96–1.11)	1.00 (0.89–1.13)	1.00 (0.83–1.13)	0.98 (0.86–1.12)
Coping	0.67 (0.31–1.42)	1.57 (0.78–3.15)	0.70 (0.27–1.78)	0.46 (0.14–1.49)	3.00 (1.01–8.94)*

*Notes*: CES-D-R = Center for Epidemiologic Studies Depression Scale-Revised; FAQ-I = functional activities questionnaire—informant-rated; MMSE = Mini-Mental State Examination; MSRS = modified social restriction scale; NPI = neuropsychiatric inventory; PAI = positive affect index; ref = reference category; RSS = relative stress scale. Data values represent rate ratios (95% confidence interval) for all measures, except for coping, for which the values represent the adjusted odds ratio (95% confidence interval). Models are adjusted for caregiver sex, caregiver age, diagnosis type, and caregiver status.

^*^Confidence intervals do not span one.

### Stress and Positive Aspects of Caregiving

The three-class solution for joint stress/PAC classes is shown in Supplementary Figure 5. Class 1 was the largest (72.2%) and had increasing stress and mid-level stable scores of PAC. Class 2 (15.2%) again had increasing levels of stress and high baseline PAC which declined slightly. Class 3 (12.5%) had increasing levels of stress, which was higher at baseline than both Class 1 and Class 2, and increasing PAC, which was very low at baseline. Characteristics of the baseline scores within each class are shown in Supplementary Table 13. Multinomial regression was conducted with Class 1 as the reference. As there were some differences in the characteristics of classes (see Supplementary Table 14), we explored whether these differences might be explained by changes in study measures over time using mixed-effects models (Supplementary Tables 15 and 16). There were few differences between classes, with the only difference being that those in Class 3 were more likely to be caring for someone with increasing severity of NPI symptoms over time.

## Discussion

To our knowledge, this is the first study to explore, in a large cohort of caregivers of community-dwelling people with dementia, longitudinal trajectories of both stress and PAC. The findings partially support our original hypotheses in that we observed changes in caregiving stress scores, although PAC scores remained stable. Different classes were identified for both measures. For stress, we identified a four-class solution, a stable class with high levels of stress, a middle class with slightly increasing levels of stress over time, a class with low levels of stress which slightly increased over time, and a small class where stress level started low but increased at a steeper rate over time. For PAC, we identified a five-class solution. There were three classes that showed little change over time. In addition, there were two small classes with changing PAC scores, one increasing and one decreasing over time. The different classes of stress and PAC were associated with factors relating to experiences of caregiving, the relationship with the person with dementia, psychological characteristics and health, and characteristics of the person with dementia. We also explored joint trajectories of stress and PAC, identifying three classes. Longitudinally, there were few differences between those classes with the exception that those in Class 3, characterized by increasing stress and PAC, were more likely to be caring for someone with increasing severity of symptoms. These findings have implications for policy and practice in terms of identifying those caregivers at risk of decline and ensuring caregivers receive adequate support.

Stress had the most noticeable changes over time. Most of the different classes had increases in stress over time with the exception of those in the High stress class, which stayed stable, possibly related to ceiling effects. Increases in caregiving stress over time are in line with previous findings on caregiving burden (van den Kieboom et al., 2020). The findings from the current study indicate that multiple factors were linked to these trajectories. It is notable that those in the High stress class were characterized by higher NPI scores (all three scales), and by variables linked to their caregiving experience, psychological health, and relationship with the person with dementia. The range of factors linked to these classes expands the findings from longitudinal studies identified by van den Kieboom et al. (2020). Those studies predominately focused on characteristics associated with the care recipient with little consideration of factors associated with the caregiver. The findings also map onto the key domains associated with caregiving burden identified by van der Lee et al. (2014). To our knowledge, only Svendsboe et al. (2018) have explored stress longitudinally, identifying differences in trajectories due to diagnosis and living situation. In the current study, the only impact of diagnosis was that those in the Low stress class, compared to the Middle stress class, were less likely to care for someone with PDD or DLB, a common factor being problems with movement in both conditions. This, to some extent, aligns with Svendsboe et al.’s (2018) findings that caregivers for people with DLB had higher stress. Apart from this finding, dementia diagnosis had no other association with class membership for either stress or PAC.

Stress did not decline over time in any of the identified classes. Conde-Sala et al. (2014) identified a class with an initially high caregiving burden which decreased over time; this was linked to a decrease in NPI scores and an improvement in caregiver mental health. Although in the current study, the Low stress class was characterized by lower NPI and depression scores this did not lead to a reduction in stress. Despite not identifying decreases in stress scores in this study, we did identify a small class with quite a steep increase in stress scores. Analyses exploring change over time indicate that this Increasing stress class also had increasing NPI scores (all three scales), and a decline in the care recipient’s functional abilities and dependence. They were more likely to show a decline in competence, relationship quality, coping, PAC, and health, with an increase in feelings of social restriction and depression. Therefore, these findings suggest that knowledge of these variables might be important in helping to develop targets for intervention.

Few studies have explored PAC longitudinally and none have examined differences in trajectories of PAC. Overall, the findings of the current study show that caregivers’ reports of PAC remained stable over time, with only two very small classes showing any change over time. This is different from Lévesque et al. (1998) who reported a nonsignificant decrease in PAC over time, though this was over a shorter follow-up period than the current study. Our finding does align with previous research around “living well” in caregivers (Clare et al., 2022) which indicated a predominantly stable trajectory over time. The stability of PAC is an interesting finding when compared to the observed increase in caregiving stress. Theoretical perspectives around positive emotions identify that their role in coping may develop over time. The broaden-and-build theory (Fredrickson, 2004) indicates that over time positive emotions broaden a person’s way of thinking and so this adaptive effect may play a greater role later on in the caregiving career. Similarly, it has been suggested that benefit-finding emerges over time in the process of adapting to adversity and, in the long term, reflects actual growth or change (Tennen & Affleck, 2002). From these models, we might have expected PAC scores to increase over time. The predominantly stable PAC scores may reflect pre-existing inner growth within caregivers and that this identification of positive experiences remained stable despite increasing stress. This was also demonstrated in the joint trajectories of stress and PAC whereby PAC scores tended to remain stable despite stress levels. There was only a very small class where a slight decline in PAC was observed. This may suggest stress and PAC involve different internal processes with the chronic stress associated with being a caregiver triggering stress-related reactions, and PAC being either a conscious or an implicit adaptational approach (Quinn et al., 2024).

Although experiences of PAC were relatively stable over time, there were differences within group membership. Those in the high PAC group were more likely to have higher self-esteem, competence, relationship quality, and coping ability at baseline. They also were less socially restricted and stressed and more likely to be male. Similarly, although the declining PAC class was very small and results should be interpreted with caution, longitudinally, they were more likely to have a steeper decline in competence, relationship quality, and coping, to become more depressed, and to rate the care recipient as more functionally impaired. These factors have been connected with PAC cross-sectionally, but to our knowledge, this is the first time they have been linked to longitudinal trajectories of PAC. The role of relationship quality supports the conceptual model of PAC by Carbonneau et al. (2010) but also highlights other domains that can influence PAC and could be targeted through intervention to help caregivers maintain PAC.

In considering the findings, the limitations of the study need to be acknowledged. Despite having a large cohort of caregivers at baseline, there was attrition at subsequent time points. Attrition is not uncommon in cohort studies and has been reported in other studies (e.g., Conde-Sala et al., 2014). Healthy caregiver survivor bias may have had an influence on the classes of trajectories extracted, meaning certain profiles of caregivers were missed. This may be particularly true for those who experienced the most stress because those who dropped out were likely to have higher stress scores. Other measures associated with dropout at T2 or T3 included providing more hours of care, being a family/friend caregiver, caring for someone with greater impairment, and having a poorer relationship quality with the person with dementia. Higher neuroticism, poorer self-esteem, greater distress at neuropsychiatric symptoms, and coping less well at T1 were associated with dropout at T2, and poorer self-rated health at T2 was associated with dropout at T3. The study predominantly involved spousal caregivers, though we identified few differences according to kin relationship within the resulting classes. There were slightly more female caregivers within the sample, but over two thirds of caregivers are females (Alzheimer’s Association, 2023). Similarly, predominantly the sample was White British, but this reflects the population in the United Kingdom accessing dementia diagnostic services (Pham et al., 2018). Measure selection was based on the findings from the literature, but we recognize that other factors, such as whether the caregiver received additional support or if the person with dementia had recently suffered from ill health, may potentially influence the caregiver’s experience. The classes extracted from the GMM-class invariant model should be interpreted with some caution; GMM is an exploratory approach and findings vary based on model specification. Although a GMM with free variances both within and across classes is optimal, to support convergence, it was necessary to constrain the intercept and slope variances to be equal across classes. However, plots of the resulting classes show clear distinctions in the patterns of trajectories. Additionally, the findings are as we might expect, particularly for the stable classes; for example, more positive scores on study measures are associated with the “low” stress class versus the “middle” stress class, and more negative scores on study measures are association with the “high” stress class. As would be expected, there is overlap between classes and, because of this, the probability of a participant being a member of each class was taken into account. Finally, some of the identified classes were small and had limited statistical power. Therefore, weaker associations may not have been identified.

## Implications and Conclusions

The findings of the study have implications for policy and practice. The provision of post-diagnostic support is fragmented and there is variation in provision within and across countries (Alzheimer’s Association, 2023). Often caregivers come into the role with no prior experience of caregiving and will need to access help from support services. Typically, caregivers are offered information and support as part of the diagnostic process, but their support needs will change over time as the person with dementia becomes more impaired. Therefore, there is a need for continual post-diagnostic support for caregivers that takes into account these changing needs. However, provision of this support is often patchy and reliant on the caregiver seeking out support. Our findings highlight that caregivers are not one homogenous group and there are differences in their experiences which point to the need for provision of more holistic interventions that can be tailored to individual needs. The findings have implications for identifying those caregivers at risk of decline, as there were some groups of caregivers that were more at risk of negative outcomes over time. For example, it is notable that the High stress class did not change over time. This suggests this group of caregivers may benefit from being identified at an earlier stage as timelier intervention with this group may have been beneficial. Often access to support is “reactive” in that caregivers access it when they are in crisis rather than it being “preventative” and put in place to prevent these crises from occurring. We also identified an Increasing stress class and a Decreasing PAC group; both were characterized by potentially modifiable variables and changes could be mitigated through post-diagnostic support interventions. Thus, identifying these caregivers at an early stage and offering interventions would potentially be beneficial. Psychosocial interventions have been found to be effective in improving caregiver outcomes (Teahan et al., 2020) and these types of interventions could be offered to caregivers. It is also important that healthcare professionals recognize that caregiver stress may change and those who appear to be coping well may experience changes in their support needs over time. Caregivers could be supported to continue identifying PAC within their role so that it does not decline. In conclusion, this study has identified differences in trajectories of caregiving stress and PAC highlighting the need to take a holistic approach to supporting caregivers.

## Supplementary Material

gbae097_suppl_Supplementary_Materials

## Data Availability

For the purpose of open access, the authors have applied a Creative Commons Attribution (CC BY) license to any Author Accepted Manuscript version arising. IDEAL data were deposited with the UK data archive in April 2020. Details of how to access the data can be found here: https://reshare.ukdataservice.ac.uk/854293/.
