# Supplementary material for: Longitudinal Trajectories of Stress and Positive Aspects of Dementia Caregiving: Findings From the IDEAL Programme
Source: J Gerontol B Psychol Sci Soc Sci. 2024 May 30;79(8):gbae097. doi: 10.1093/geronb/gbae097 (PMC11226992; doi:10.1093/geronb/gbae097)

**Supplementary information**

**Description of measures**

**Demographic measures:** Measures included caregiver status (spouse/partner and family/friend), caregiver sex, caregiver age, living situation (lives with care recipient and does not live with care recipient), and dementia subtype of the care recipient (Alzheimer’s disease, vascular dementia, mixed Alzheimer’s disease and vascular dementia, frontotemporal dementia, Parkinson’s disease dementia, dementia with Lewy bodies, and other/unspecified). Caregiver education was classified into four groups; no qualifications, school leaving certificate at age 16 (GCSE or equivalent), school leaving certificate at age 18 (A level or equivalent), and university level education. Socioeconomic status was classified based on Office for National Statistics (Office for National Statistics, 2010) classifications using the National Statistics Socio-Economic Classification three categories. The three class NS-SEC groups participants into: High (Higher managerial, administrative, and professional occupations), Middle (Intermediate occupations) and Low (Routine and manual occupations). People who never worked were coded as missing. The number of hours spent caregiving per day were categorised into under 1 hour, 1-10 hours, and over 10 hours.

**Measures about the caregiver**

***Experiences of caregiving.***

*Relative Stress Scale (RSS).*

This is a 15-item measure assessing the degree of distress and social upset experienced by a relative as a result of caring for a person with physical or behavioural difficulties (example item: Do you ever feel frustrated with your relative/friend?). Scores range from 0-60 with a higher score indicates more severe stress (Greene et al., 1982).

*Positive Aspects of Caregiving (PAC).*

This is a nine-item questionnaire that investigates positive aspects of being a caregiver such as whether providing help made the caregiver feel useful (example item: Providing help to my relative/friend has made me feel appreciated). Scores range 9-45, with higher values indicating increased positive aspects (Tarlow et al., 2004).

*The Modified Social Restriction Scale (MSRS).*

Two items were used to explore whether the caregiver felt there was anyone else who could help the caregiver if s/he was ill or needed some respite from caregiving. A higher score indicates more difficulties (Balducci et al., 2008).

*Caregiver competency.*

This was measured using three items assessing the extent to which caregivers of people with dementia feel they are doing an adequate job as a caregiver (example item: How often do you feel that you are doing a good job as a carer?). Possible scores range from 0-12, with higher scores indicating greater competence (Robertson et al., 2007);

*Coping.*

This was measured using the single item “Do you think you cope well as a caregiver?”(McKee et al., 2003), with answers ranging from 1 (never) to 4 (always). For analyses, the responses never/sometimes and often/always were combined.

***Psychological characteristics and health.***

*Neuroticism.*

This was measured using the mini-IPIP neuroticism measure (example item: I seldom feel blue). Possible scores range from 4-20, with a higher score indicates higher rates of neuroticism ( Donnellan et al., 2006).

*Self-esteem (RSE)*

This was measured using the ten-item Rosenberg Self-Esteem Scale (example item: I feel that I’m a person of worth, at least on an equal plane with others). Possible scores range from 10-40 with higher scores indicate greater self-esteem (Rosenberg, 1965).

*Depression.*

The 20-item Center for Epidemiologic Studies Depression Scale-Revised (CESD-R) was used to measure depression (example item: I felt sad). Possible scores range from 0-60, with higher scores indicative of more depressive symptoms (Eaton et al., 2004).

***Relationship with person with dementia.***

*Relationship quality.*

The five-item Positive Affect Index provides a measure of current relationship quality (example item: How often do you and your relative friend do things together). Possible scores range 5-30, with a higher score indicates better relationship quality (Bengtson & Schrader, 1982).

***Physical Health.***

*Health.*

Self-rated health was assessed using a single-item ‘overall, how would you rate your health in the past 4 weeks?’ that ranges from 1 (very poor) to 6 (excellent). Higher scores indicate higher subjective health (Bowling, 2005)

**Measures about the care-recipient**

**Functional ability.**

Caregivers completed the modified 11-item Functional Activities Questionnaire (FAQ; Martyr et al., 2012; Pfeffer et al., 1982) to rate the current functional ability of the care recipient (example item: Can your relative/friend shop alone for clothes, household necessities and groceries?) Possible scores ranged from 0-33 with higher scores indicating greater functional impairment.

Caregivers completed the Dependence Scale, a 13-item questionnaire, was used to measure the amount of assistance needed by the person with dementia, to rate dependence (example item: Does your relative/friend need to be tube fed?). Possible scores ranged from 0-15. A higher score indicated greater functional impairment. (Brickman et al., 2002)

**Cognition.**

Cognition of the person with dementia was assessed using the Mini-Mental State Examination (MMSE: Folstein et al., 1975). Scores for the MMSE range between 0 and 30, and a higher score indicates better cognition.

**Neuropsychiatric symptoms.**

Neuropsychiatric symptoms were assessed using the 12-item Neuropsychiatric Inventory-Questionnaire (Kaufer, 2000). Item wording was adapted from the 2008 version used in the US National Alzheimer’s Coordinating Center (Wood et al., 2000); this was to facilitate self-completion by the caregiver (Example item: Is your relative/friend stubborn and resistive to help from others?). Scores range between 0 and 12. If a symptom was present two sub-questions were asked concerning severity of the symptom (scores range between 0 and 36) and how distressing the caregiver found that symptom (scores range between 0 and 72). For all three scores, higher scores indicate more symptoms, higher symptom severity, or greater distress at the symptom.

**References**

Balducci, C., Mnich, E., McKee, K. J., Lamura, G., Beckmann, A., Krevers, B., Wojszel, Z. B., Nolan, M., Prouskas, C., & Bień, B. (2008). Negative impact and positive value in caregiving: Validation of the COPE index in a six-country sample of carers. *The Gerontologist*, *48*(3), 276-286. https://doi.org/10.1093/geront/48.3.276

Bengtson, V. L., & Schrader, S. S. (1982). Parent-child relations. In *Research instruments in social gerontology: Social roles and social participation* (Vol. 2, pp. 115-185). University of Minnesota Press. .

Bowling, A. (2005). Just one question: if one question works, why ask several? *Journal of Epidemiology and Community Health*, *59*(5), 342-345. https://doi.org/10.1136/jech.2004.021204

Brickman, A. M., Riba, A., Bell, K., Marder, K., Albert, M., Brandt, J., & Stern, Y. (2002). Longitudinal assessment of patient dependence in Alzheimer disease. *Archives of Neurology*, *59*(8), 1304-1308. https://doi.org/https://doi.org/10.1001/archneur.59.8.1304

Donnellan, M. B., Oswald, F. L., Baird, B. M., & Lucas, R. E. (2006). The mini-IPIP scales: tiny-yet-effective measures of the Big Five factors of personality. *Psychological Assessment*, *18*(2), 192-203. https://doi.org/https://doi.org/10.1037/1040-3590.18.2.192

Eaton, W. W., Smith, C., Ybarra, M., Muntaner, C., & Tien, A. (2004). Center for Epidemiologic Studies Depression Scale: review and revision (CESD and CESD-R). In M. E. Maruish (Ed.), *The Use of Psychological Testing for Treatment Planning and Outcomes Assessment* (3rd ed., Vol. Volume 3: Instruments for Adults, pp. 363-377). Lawrence Erlbaum.

Greene, J. G., Smith, R., Gardiner, M., & Timbury, G. C. (1982). Measuring behavioural disturbance of elderly demented patients in the community and its effects on relatives: A factor analytic study. *Age and Ageing*, *11*(2), 121-126. https://doi.org/10.1093/ageing/11.2.121

Kaufer, D. I. (2000). Validation of the NPI-Q, a Brief Clinical Form of the Neuropsychiatric Inventory. *Journal of Neuropsychiatry*, *12*(2), 233-239. https://doi.org/https://doi.org/10.1176/appi.neuropsych.12.2.233

Martyr, A., Clare, L., Nelis, S. M., Markova, I. S., Roth, I., Woods, R. T., Whitaker, C. J., & Morris, R. G. (2012). Verbal fluency and awareness of functional deficits in early-stage dementia. *Clinical Neuropsychologist*, *26*(3), 501-519. https://doi.org/https://doi.org/10.1080/13854046.2012.665482

McKee, K., Philp, I., Lamura, G., Prouskas, C., Öberg, B., Krevers, B., Spazzafumo, L., Bien, B., Parker, C., & Nolan, M. (2003). The COPE index-a first stage assessment of negative impact, positive value and quality of support of caregiving in informal carers of older people. *Aging & Mental Health*, *7*(1), 39-52. https://doi.org/10.1080/1360786021000006956

Office for National Statistics. (2010). *Office for National Statistics: Standard occupational classification 2010. Volume 3. The national statistics socioeconomic classification: (Rebased on the SOC2010) User Manual* (Vol. 3). Palgrave Macmillan.

Pfeffer, R. I., Kurosaki, T. T., Harrah, C. H., Chance, J. M., & Filos, S. (1982). Measurement of Functional Activities in Older Adults in the Community. *Journal of Gerontology*, *37*(3), 323-329. https://doi.org/https://doi.org/10.1093/geronj/37.3.323

Robertson, S. M., Zarit, S. H., Duncan, L. G., Rovine, M. J., & Femia, E. E. (2007). Family caregivers’ patterns of positive and negative affect. *Family Relations*, *56*(1), 12-23. https://doi.org/10.1111/j.1741-3729.2007.00436.x

Rosenberg, M. (1965). *Society and the adolescent self-image.* Princeton University Press.

Tarlow, B. J., Wisniewski, S. R., Belle, S. H., Rubert, M., Ory, M. G., & Gallagher-Thompson, D. (2004). Positive aspects of caregiving: contributions of the REACH project to the development of new measures for Alzheimer's caregiving. *Research on Aging*, *26*(4), 429-453. https://doi.org/10.1177/0164027504264493

Wood, S., Cummings, J. L., Hsu, M. A., Barclay, T., Wheatley, M. V., Yarema, K. T., & Schnelle, J. F. (2000). The use of the neuropsychiatric inventory in nursing home residents. Characterization and measurement. *American Journal of Geriatric Psychiatry*, *8*(1), 75-83. https://doi.org/10.1097/00019442-200002000-00010

**Growth Mixture Model selection**

Two growth mixture models with varying levels of restrictions were tested; the CMM-CI (class-invariant) constrains the variances of the global growth factors across classes to be equal, and the CMM-CV (class-varying) freely estimates all the variances of the global growth factors (intercept and slope) across all classes. A frequent problem with growth mixture models is non-convergence and local solutions (Hipp & Bauer 2006), with more complex models involving free variances more likely to have convergence difficulties (Grimm & Ram 2009). Between 1 and 5 class solutions were tested for each assumption, with 1000 random starts and 20 iterations for each model in order to avoid local solutions. Following successful convergence the optimal number of distinct trajectories was determine using the Bayesian Information Criterion (BIC), sample size adjusted BIC (ssa-BIC), the Lo-Mendell-Rubin likelihood ratio test (LMR-LRT), and the bootstrapped likelihood ratio test (BLRT) which provide between model comparisons (k vs k-1), and entropy (Jung & Wickrama 2008, Nylund 2007, Tein 2013). Entropy is a standardised index of a model-based classification accuracy based on the average posterior probability, with higher values indicating clearer class separation (Muthen 2004). Substantive criteria were based on a class size greater than 1% and theoretical and practical interpretability of the classes. Upon finding the optimal solution, the model was repeated with double the number of starts and iterations to ensure a global solution.

Due to their person-centred approach, GMMs allow for examination of predictors of class membership (Wickrama et al., 2016). The categorical latent class is related to the covariates by way of multinomial logistic regression which assigns each individual fractionally to all classes using posterior probabilities. Predictors of class were examined using the ‘3-step’ approach in Mplus (R3STEP) in order to protect the latent class structure from influences of the covariates (Asparouhov & Muthen, 2013; Vermunt, 2010).

*Stress model:*

Two models were tested as described above: GMM-CI and GMM-CV. One to 5 class solutions of each model were conducted, and model fit indices and class sizes are displayed in Supplementary Table 1 for those models that converged. The resulting trajectories for each solution are shown in Supplementary Figure 1. The GMM-CI models are less computationally intensive, and all classes ran with no convergence issues. The GMM-CV model with free intercepts and slopes experienced convergence issues for solutions with more than 2 classes. Given all available information including model fit indices, interpretability, and theoretical considerations, the 4-class solution was selected.

*PAC model:*

Again 1 to 5 class solutions were tested for the GMM-CI and GMM-CV (Supplementary Table 3 and Supplementary Figure 2. All GMM-CI models ran with no convergence issues. The GMM-CV model with free intercepts and slopes experienced convergence issues for solutions greater than 2 classes. Given all available information including model fit indices, interpretability, and theoretical considerations, the 5-class solution was selected.

**References**

Asparouhov, T., & Muthen, B. (2013). Auxiliary variables in mixture modeling: a 3-step approach using Mplus. Mplus Web Notes: No. 15. Version 6.

Grimm, K. J., & Ram, N. (2009). Non-linear growth models in Mplus and SAS. Structural Equation Modeling, 16(4), 676-701. https://doi.org/10.1080/10705510903206055

Hipp, J. R., & Bauer, D. J. (2006). Local solutions in the estimation of growth mixture models. Psychological Methods, 11(1), 36-53. https://doi.org/10.1037/1082- 989X.11.1.36

Jung, T., & Wickrama, K. A. S. (2008). An introduction to latent class growth analysis and growth mixture modeling. Social and Personality Psychology Compass, 2(1), 302- 317. <https://doi.org/10.1111/j.1751-9004.2007.00054.x>

Muthén, B. (2004). Latent variable analysis: growth mixture modeling and related techniques for longitudinal data. In D. Kaplan (Ed.), Handbook of quantitative methodology for the social sciences. Sage Publications. <https://doi.org/10.4135/9781412986311.n19>

Nylund, K. L., Asparouhov, T., & Muthen, B. (2007). Deciding on the number of classes in latent class analysis and growth mixture modeling: a Monte Carlo simulation study. 43 Structural Equation Modeling, 14(4), 535-569. <https://doi.org/10.1080/10705510701575396>

Tein, J. Y., Coxe, S., & Cham, H. (2013). Statistical power to detect the correct number of classes in latent profile analysis. Structural Equation Modeling, 20(4), 640-657. https://doi.org/10.1080/10705511.2013.824781

Vermunt, J. K. (2010). Latent class modeling with covariates: two improved three-step approaches. Political Analysis, 18(4), 450-469. https://doi.org/10.1093/pan/mpq025

Wickrama, K. A. S., Lee, T. K., O'Neal, C. W., & Lorenz, F. O. (2016). Higher-order growth curves and mixture modeling with Mplus. A practical Guide. Routledge. <https://doi.org/10.4324/9781315642741>

**Supplementary Table 1. Growth mixture model selection for classes of stress**

| **GMM-CI** | **1 class** | **2 class** | **3 class** | **4 class** | **5 class** |
| --- | --- | --- | --- | --- | --- |
| LL | -9067 | -9053 | -9034 | -9012 | -8996 |
| BIC | 18191 | 18184 | 18168 | 18145 | 18133 |
| ssa-BIC | 18166 | 18149 | 18123 | 18091 | 18069 |
| Entropy | 1 | 0.492 | 0.491 | 0.679 | 0.718 |
| Adj LMR-LRT (p) | - | 27.31 (0.106) | 45.05 (0.000) | 39.87 (0.066) | 33.42 (0.001) |
| BLRT (p) | - | 28.60 (0.000) | 44.02 (0.000) | 41.77 (0.000) | 34.99 (0.000) |
| Classes 1 | 100% | 68.2% | 38.2% | 8.3% | 38.8% |
| 2 |  | 31.7% | 45.7% | 46.1% | 7.6% |
| 3 |  |  | 16.1% | 39.5% | 0.4% |
| 4 |  |  |  | 6.1% | 7.3% |
| 5 |  |  |  |  | 45.9% |
| **GMM-CV** | **1 class** | **2 class** | **3 class ^a^** | **4 class ^a^** | **5 class ^a^** |
| LL | -9633 | -9038 |  | - | - |
| BIC | 19322 | 18170 |  |  |  |
| ssa-BIC | 19297 | 18128 |  |  |  |
| Entropy | 1 | 0.591 |  |  |  |
| Adj LMR-LRT (p) | - | 55.49 (0.001) |  |  |  |
| BLRT (p) | - | 57.06 (0.000) |  |  |  |
| Classes 1 | 100% | 18.4% |  |  |  |
| 2 |  | 81.6% |  |  |  |

*Note.* GMM-CI, growth mixture model – class invariant (variances and covariances are fixed between classes); GMM-CV, growth mixture modelling – class varying (variances and covariances are freely estimated); LL, loglikelihood; BIC, Bayesian Information Criterion; ssa-BIC, sample-size adjusted BIC; adj LMR-LRT , LoMendell-Rubin adjusted Likelihood Ratio Test; BLRT , Bootstrap Likelihood Ratio Test; C1-C5, class 1-class 5. ^a^ inadmissible solution

**Supplementary Table 2. Average latent class probabilities for most likely latent class membership (row) by latent class (column) for the 4-class model of stress**

|  | **Class 1. High** | **Class 2. Middle** | **Class 3. Low** | **Class 4. Increasing** |
| --- | --- | --- | --- | --- |
| Class 1. High | 0.828 | 0.172 | 0.000 | 0.000 |
| Class 2. Middle | 0.053 | 0.825 | 0.101 | 0.021 |
| Class 3. Low | 0.000 | 0.109 | 0.829 | 0.061 |
| Class 4. Increasing | 0.000 | 0.055 | 0.139 | 0.806 |

*Note.* Each column represents classification probabilities (averages of the individual probabilities in each class) for the 4-class solution of the estimated GMM-CI, and each row represents the classification probabilities for the most likely class. If classes were perfectly separated, the diagonal components would be 1, and the off diagonals would be 0.

**Supplementary Table 3. Growth mixture model selection for positive aspects of caregiving**

|  | **1 class** | **2 class** | **3 class** | **4 class** | **5 class** |
| --- | --- | --- | --- | --- | --- |
| LL | -8656 | -8642 | -8594 | -8587 | -8577 |
| BIC | 17368 | 17362 | 17287 | 17294 | 17296 |
| ssa-BIC | 17342 | 17327 | 17243 | 17240 | 17233 |
| Entropy | 1 | 0.797 | 0.819 | 0.826 | 0.787 |
| Adj LMR-LRT (p) | - | 25.64 (0.091) | 92.03 (0.000) | 14.08 (0.089) | 17.57 (0.092) |
| BLRT (p) | - | 26.85 (0.000) | 96.36 (0.000) | 14.74 (0.000) | 18.40 (0.000) |
| Classes 1 | 100% | 8.1% | 71.7% | 15.8% | 15.2% |
| 2 |  | 91.9% | 15.8% | 71.4% | 67.6% |
| 3 |  |  | 12.5% | 2.8% | 9.3% |
| 4 |  |  |  | 10.0% | 3.4% |
| 5 |  |  |  |  | 4.5% |
| **GMM-CV** | **1 class** | **2 class** | **3 class ^a^** | **4 class ^a^** | **5 class ^a^** |
| LL | -8656 | -8631 |  | - | - |
| BIC | 17368 | 17353 |  |  |  |
| ssa-BIC | 17342 | 17312 |  |  |  |
| Entropy | 1 | 0.906 |  |  |  |
| Adj LMR-LRT (p) | - | 48.58 (0.001) |  |  |  |
| BLRT (p) | - | 49.95 (0.000) |  |  |  |
| Classes 1 | 100% | 94.0% |  |  |  |
| 2 |  | 6.0% |  |  |  |

*Note.* GMM-CI, growth mixture model – class invariant (variances and covariances are fixed between classes); GMM-CV, growth mixture modelling – class varying (variances and covariances are freely estimated); LL, loglikelihood; BIC, Bayesian Information Criterion; ssa-BIC, sample-size adjusted BIC; adj LMR-LRT , LoMendell-Rubin adjusted Likelihood Ratio Test; BLRT , Bootstrap Likelihood Ratio Test; C1-C5, class 1-class 5. ^a^ inadmissible solution

**Supplementary Table 4. Average latent class probabilities for most likely latent class membership (row) by latent class (column) for the 5-class model of positive aspects of caregiving**

|  | **Class 1. High** | **Class 2. Middle** | **Class 3. Low** | **Class 4. Increasing** | **Class 5. Decreasing** |
| --- | --- | --- | --- | --- | --- |
| Class 1. High | 0.872 | 0.118 | 0.000 | 0.000 | 0.010 |
| Class 2. Middle | 0.041 | 0.899 | 0.020 | 0.004 | 0.036 |
| Class 3. Low | 0.000 | 0.098 | 0.754 | 0.133 | 0.016 |
| Class 4. Increasing | 0.000 | 0.051 | 0.150 | 0.799 | 0.000 |
| Class 5. Decreasing | 0.018 | 0.183 | 0.012 | 0.000 | 0.787 |

*Note.* Each column represents classification probabilities (averages of the individual probabilities in each class) for the 5-class solution of the estimated GMM-CI, and each row represents the classification probabilities for the most likely class. If classes were perfectly separated, the diagonal components would be 1, and the off diagonals would be 0.

**Supplementary Table 5. Growth mixture model selection for classes of joint stress/PAC (GMM-CI)**

|  | **2 class** | **3 class** | **4 class** | **5 class** | **6 class** |
| --- | --- | --- | --- | --- | --- |
| Entropy | 0.900 | 0.891 | 0.853 | 0.855 | 0.833 |
| BIC | 35445 | 35389 | 35403 | 35405 | 35419 |
| ssBIC | 35372 | 35300 | 35298 | 35284 | 35282 |
| Class 1 | 2.9% | 72.2% | 72.2% | 12.5% | 68.1% |
| Class 2 | 97.1% | 15.2% | 12.5% | 0.4% | 1.9% |
| Class 3 |  | 12.5% | 14.9% | 1.9% | 1.1% |
| Class 4 |  |  | 0.3% | 13.2% | 0.5% |
| Class 5 |  |  |  | 72% | 10.5% |
| Class 6 |  |  |  |  | 13.3% |

**Supplementary Table 6. Average latent class probabilities for most likely latent class membership (row) by latent class (column) for the 3-class model of joint stress/PAC**

|  | **Class 1. High** | **Class 2. Middle** | **Class 3. Low** |
| --- | --- | --- | --- |
| Class 1. | 0.958 | 0.025 | 0.017 |
| Class 2. | 0.200 | 0.800 | 0.000 |
| Class 3. | 0.138 | 0.000 | 0.862 |

*Note.* Each column represents classification probabilities (averages of the individual probabilities in each class) for the 3-class solution of the estimated GMM-CI, and each row represents the classification probabilities for the most likely class. If classes were perfectly separated, the diagonal components would be 1, and the off diagonals would be 0.

**Supplementary Table 7. Characteristics of the caregivers, stratified by those that remained in the study and those who left the study at the next time point**

|  | **T1 (baseline)** | | | | **T2** | | | |
| --- | --- | --- | --- | --- | --- | --- | --- | --- |
|  | **Total**  **(n=1203)** | **Remained in study at T2**  **(n=911)** | **Did not remain in study at T2**  **(n=292)** | **P-value** | **Total**  **(n=917)** | **Remained in study at T3**  **(n=680)** | **Did not remain in study at T3**  **(n=237)** | **P-value** |
| Caregiver status (n, %) |  |  |  | <0.001 |  |  |  | 0.031 |
| Spouse/partner | 997 (82.9%) | 781 (85.7%) | 216 (74.0%) |  | 782 (85.3%) | 590 (86.8%) | 192 (81.0%) |  |
| Family/friend | 206 (17.1%) | 130 (14.3%) | 76 (26.0%) |  | 135 (14.7%) | 90 (13.2%) | 45 (19.0%) |  |
| Diagnosis (n, %) |  |  |  | 0.143 |  |  |  | 0.133 |
| AD | 678 (56.4%) | 521 (57.2%) | 157 (53.8%) |  | 517 (56.4%) | 397 (58.4%) | 120 (50.6%) |  |
| VaD | 132 (11.0%) | 89 (9.8%) | 43 (14.7%) |  | 84 (9.2%) | 59 (8.7%) | 25 (10.5%) |  |
| Mixed AD/VaD | 245 (20.4%) | 194 (21.3%) | 51 (17.5%) |  | 205 (22.4%) | 146 (21.5%) | 59 (24.9%) |  |
| FTD | 43 (3.6%) | 32 (3.5%) | 11 (3.8%) |  | 35 (3.8%) | 29 (4.3%) | 6 (2.5%) |  |
| PDD/DLB | 76 (6.3%) | 52 (5.7%) | 24 (8.2%) |  | 57 (6.2%) | 35 (5.1%) | 22(9.3%) |  |
| Unspecified/Other | 29 (2.4%) | 23 (2.5%) | 6 (2.1%) |  | 19 (2.1%) | 14 (20.6%) | 5 (2.1%) |  |
| Caregiver age (years)  (mean, sd, N) | 69.3 (11.0), 1203 | 69.5 (10.5), 911 | 69.0 (12.3), 292 | 0.554 | 70.5 (10.5), 914 | 70.3 (10.3), 680 | 70.9 (11.2), 234 | 0.249 |
| Caregiver sex (n, %) |  |  |  | 0.827 |  |  |  | 0.256 |
| Male | 371 (30.8%) | 283 (31.1%) | 88 (30.1%) |  | 287 (30.3%) | 220 (32.4%) | 67 (28.3%) |  |
| Female | 832 (69.2%) | 628 (68.9%) | 204 (69.9%) |  | 630 (68.7%) | 460 (67.6%) | 170 (71.7%) |  |
| Caregiver education (n, %) |  |  |  | 0.293 |  |  |  | 0.837 |
| No qualifications | 265 (22.2%) | 197 (21.7%) | 69 (23.9%) |  | 195 (21.6%) | 141 (21.0%) | 54 (23.4%) |  |
| School leaving certificate at 16 | 266 (22.2%) | 205 (22.6%) | 61 (21.1%) |  | 205 (22.7%) | 154 (22.9%) | 52 (22.5%) |  |
| School leaving certificate at 18 | 362 (30.3%) | 265 (29.2%) | 96 (33.2%) |  | 263 (29.1%) | 194 (28.9%) | 68 (29.4%) |  |
| University | 303 (25.3%) | 240 (26.5%) | 63 (21.8%) |  | 240 (26.6%) | 183 (27.2%) | 57 (24.7%) |  |
| Caregiver socioeconomic status (n, %) |  |  |  | 0.676 |  |  |  | 0.301 |
| High | 488 (42.1%) | 375 (42.7%) | 113 (40.2%) |  | 367 (42.5%) | 273 (42.5%) | 94 (42.3%) |  |
| Middle | 415 (35.8%) | 314 (35.7%) | 101 (35.9%) |  | 308 (35.6%) | 236 (36.8%) | 72 (32.4%) |  |
| Low | 257 (22.2%) | 190 (21.6%) | 67 (23.8%) |  | 189 (21.9%) | 133 (20.7%) | 56 (25.2%) |  |
| Caregiver hours per day (n, %) |  |  |  | 0.027 |  |  |  | 0.046 |
| Under 1 hour | 269 (22.6%) | 209 (23.2%) | 60 (20.6%) |  | 148 (16.6%) | 122 (18.3%) | 26 (11.4%) |  |
| 1-10 hours | 465 (39.0%) | 365 (40.6%) | 100 (34.4%) |  | 347 (38.8%) | 250 (37.5%) | 97 (42.5%) |  |
| 10+ hours | 457 (38.4%) | 326 (36.2%) | 131 (45.0%) |  | 399 (44.6%) | 294 (44.1%) | 105 (46.1%) |  |
| *Study measures* |  |  |  |  |  |  |  |  |
| RSS  (mean (sd), N) | 18.9 (9.8), 1120 | 18.3 (9.6), 864 | 21.0 (10.5), 256 | <0.001 | 21.5 (10.1), 857 | 20.8 (9.9), 653 | 23.5 (10.5), 204 | <0.001 |
| PAC (mean (sd), N) | 28.3 (7.4), 1155 | 29.2 (7.4), 892 | 28.8 (7.5), 263 | 0.128 | 28.2 (7.7), 862 | 28.3 (7.5), 659 | 27.8 (8.1), 203 | 0.242 |
| Neuroticism (miniIPIP)  (mean (sd), N) | 10.9 (3.2), 1165 | 10.7 (3.1), 895 | 11.3 (3.1), 270 | 0.008 | - | - | - | - |
| RSE  (mean (sd), N) | 31.2 (4.5), 1140 | 31.3 (4.5), 880 | 30.7 (4.6), 260 | 0.034 | - | - | - | - |
| MMSE  (mean (sd), N) | 23.1 (3.7), 1202 | 23.3 (3.6), 910 | 22.5 (3.8), 292 | <0.001 | 21.5 (5.0), 896 | 22.0 (4.8), 665 | 20.0 (5.4), 231 | <0.001 |
| NPI distress  (mean (sd), N) | 6.1 (6.3), 999 | 5.6 (6.0), 769 | 7.5 (7.1), 230 | <0.001 | 6.6 (6.7), 805 | 6.4 (6.4), 601 | 7.3 (7.2), 204 | 0.124 |
| NPI severity  (mean (sd), N) | 5.7 (4.8), 1099 | 5.4 (4.7), 843 | 6.7 (5.2), 256 | <0.001 | 6.1 (5.1), 867 | 5.8 (4.9), 647 | 6.7 (5.6), 220 | 0.042 |
| NPI symptoms  (mean (sd), N) | 3.5 (2.5), 1136 | 3.4 (2.4), 873 | 4.0 (2.5), 263 | <0.001 | 3.7 (2.5), 900 | 3.6 (2.4), 671 | 4.0 (2.8), 229 | 0.106 |
| Competence  (mean (sd), N) | 9.2 (1.7), 1158 | 9.2 (1.7), 892 | 9.0 (1.7), 266 | 0.068 | 9.1 (1.6), 865 | 9.1 (1.6), 656 | 8.9 (1.7), 209 | 0.275 |
| PAI current  (mean (sd), N) | 23.2 (4.7), 1170 | 23.5 (4.6), 896 | 22.4 (4.9), 274 | <0.001 | 22.4 (4.8), 865 | 22.6 (4.6), 662 | 21.7 (5.3), 203 | 0.020 |
| Self-rated health  (mean (sd), N) | 4.1 (1.1), 1189 | 4.1 (1.1), 900 | 4.0 (1.1), 289 | 0.150 | 3.9 (1.1), 878 | 4.0 (1.1), 667 | 3.7 (1.1), 211 | 0.007 |
| Coping (n, %)  Never/sometimes  Often/always | 290 (25.0%)  872 (75.0%) | 208 (23.3%)  686 (76.7%) | 82 (30.6%)  186 (69.4%) | 0.019 | 283 (32.5%)  587 (67.5%) | 213 (32.3%)  447 (67.7%) | 70 (33.3%)  140 (66.7%) | 0.800 |
| CESD-R  (mean (sd), N) | 7.1 (8.0), 1124 | 6.9 (7.8), 870 | 7.8 (8.7), 254 | 0.082 | 8.3 (9.1), 838 | 8.1 (8.8), 641 | 9.0 (10.0), 197 | 0.246 |
| MSRS  (mean (sd), N) | 3.5 (1.4), 1153 | 3.5 (1.4), 890 | 3.4 (1.4), 263 | 0.152 | 3.7 (1.4), 862 | 3.7 (1.4), 655 | 3.8 (1.4), 207 | 0.187 |
| FAQ (informant rated)  (mean (sd), N) | 17.6 (4.6), 1111 | 16.9 (8.5), 857 | 19.7 (8.7), 254 | <0.001 | 20.4 (8.5), 874 | 19.6 (8.6), 653 | 22.6 (7.9), 221 | <0.001 |
| Dependence (informant rated)  (mean (sd), N) | 5.6 (2.6), 1123 | 5.4 (2.5), 864 | 6.1 (2.8), 259 | <0.001 | 6.2 (2.9), 875 | 6.0 (2.8), 654 | 6.9 (3.1), 221 | <0.001 |

*Note.* Unpaired t-tests were used to compare continuous variables and Chi-squared tests to compare categorical variables between the two groups (remained in study vs did not remain in study) between T1 and T3.

AD, Alzheimer’s disease; VaD, vascular dementia; FTD, frontotemporal dementia; PDD, Parkinson’s disease dementia; DLB, dementia with Lewy bodies; RSS, Relative Stress Scale; PAC, positive aspects of caregiving; RSE, Rosenberg Self-Esteen Scale; MMSE, mini-mental state examination; NPI, neuropsychiatric inventory; PAI, positive affect index; CESD_R, Center for Epidemiologic Studies Depression Scale-Revised; MSRS, modified social restriction scale; FAQ-I, functional activities questionnaire – informant-rated.

**Supplementary Table 8. Characteristics of the cohort across the three timepoints**

|  | **T1 (n=1203)** | **T2 (n=917)** | | **T3 (n=699)** | |
| --- | --- | --- | --- | --- | --- |
| Caregiver status (n, %) |  |  | |  | |
| Spouse/partner | 997 (82.9%) | 782 (85.3%) | | 603 (86.3%) | |
| Family/friend | 206 (17.1%) | 135 (14.7%) | | 96 (13.7%) | |
| Living situation (n, %) |  |  | |  | |
| Lives with care recipient | 1054 (87.6%) | 822 (89.6%) | | 628 (89.8%) | |
| Does not live with care recipient | 149 (12.4%) | 95 (10.4%) | | 71 (10.2%) | |
| Diagnosis (n, %) |  |  | |  | |
| AD | 678 (56.4%) | 517 (56.4%) | | 405 (57.9%) | |
| VaD | 132 (11.0%) | 84 (9.2%) | | 64 (9.2%) | |
| Mixed AD/VaD | 245 (20.4%) | 205 (22.4%) | | 147 (21.0%) | |
| FTD | 43 (3.6%) | 35 (3.8%) | | 29 (4.1%) | |
| PDD/DLB | 76 (6.3%) | 57 (6.2%) | | 39 (5.6%) | |
| Unspecified/Other | 29 (2.4%) | 19 (2.1%) | | 15 (2.1%) | |
| Caregiver age (years) (mean, sd, missing) | 69.3 (11.0) | 70.5 (10.5) | | 71.2 (10.3) | |
| Caregiver sex/care recipient sex (n, %) |  |  | |  | |
| Female/male | 701 (58.3%) | 538 (58.8%) | | 407 (58.2%) | |
| Male/female | 358 (29.8%) | 280 (30.6%) | | 218 (31.2%) | |
| Female/female | 131 (10.9%) | 90 (9.8%) | | 68 (9.7%) | |
| Male/male | 13 (1.1%) | 7 (0.8%) | | 6 (0.9%) | |
| Caregiver education (n, %) |  |  | |  | |
| No qualifications | 265 (22.2%) | 195 (21.6%) | | 144 (20.9%) | |
| School leaving certificate at 16 | 266 (22.2%) | 205 (22.7%) | | 154 (22.4%) | |
| School leaving certificate at 18 | 362 (30.3%) | 263 (29.1%) | | 203 (29.5%) | |
| University | 303 (25.3%) | 240 (26.6%) | | 187 (27.2%) | |
| Missing | 7 | 14 | | 11 | |
| Caregiver socioeconomic status (n, %) |  |  | |  | |
| High | 488 (42.1%) | 367 (42.5%) | | 281 (42.8%) | |
| Middle | 415 (35.8%) | 308 (35.6%) | | 242 (36.8%) | |
| Low | 257 (22.2%) | 189 (21.9%) | | 134 (20.4%) | |
| Missing | 43 | 53 | | 42 | |
| Caregiver hours per day (n, %) |  |  | |  | |
| Under 1 hour | 269 (22.6%) | 148 (16.6%) | | 84 (12.2%) | |
| 1-10 hours | 465 (39.0%) | 347 (38.8%) | | 277 (40.0%) | |
| 10+ hours | 457 (38.4%) | 399 (44.6%) | | 330 (47.8%) | |
| Missing | 12 | 23 | | 8 | |
| *Study measures* |  |  | |  | |
| RSS  (mean (sd), missing) | 18.9 (9.8), n=83 | 21.5 (10.1), n=60 | | 23.2 (10.3), n=42 | |
| PAC  (mean (sd), missing) | 28.3 (7.4), n=48 | 28.2 (7.7), n=55 | | 27.9 (7.8), n=36 | |
| Neuroticism (miniIPIP)  (mean (sd), missing) | 10.9 (3.2), n=38 | - | | - | |
| RSE  (mean (sd), missing) | 31.2 (4.5), n=63 | - | | - | |
| MMSE  (mean (sd), missing) | 23.1 (3.7), n=1 | 21.5 (5.0), n=21 | | 20.4 (6.2), n=46 | |
| NPI distress  (mean (sd), missing) | 6.1 (6.3), n=204 | 6.6 (6.7), n=112 | | 7.6 (7.1), n=89 | |
| NPI severity  (mean (sd), missing) | 5.7 (4.8), n=104 | 6.1 (5.1), n=50 | | 6.8 (5.3), n=48 | |
| NPI symptoms  (mean (sd), missing) | 3.5 (2.5), n=67 | 3.7 (2.5), n=17 | | 4.1 (2.6), n=24 | |
| Competence  (mean (sd), missing) | 9.2 (1.7), n=45 | 9.1 (1.6), n=52 | | 9.0 (1.6), n=29 | |
| PAI current  (mean (sd), missing) | 23.2 (4.7), n=33 | 22.4 (4.8), n=52 | | 22.1 (4.8), n=34 | |
| Self-rated health  (mean (sd), missing) | 4.1 (1.1), n=14 | 3.9 (1.1), n=39 | | 3.9 (1.1), n=20 | |
| Coping (n, %)  Never/sometimes  Often/always  Missing | 290 (25.0%)  872 (75.0%)  41 | 283 (32.5%)  587 (67.5%)  47 | | 226 (33.6%)  447 (66.4%)  26 | |
| CESD-R  (mean (sd), missing) | 7.1 (8.0), n=79 | 8.3 (9.1), n=79 | | 8.8 (9.7), n=51 | |
| MSRS  (mean (sd), missing) | 3.5 (1.4), n=50 | | 3.7 (1.4), n=55 | | 3.8 (1.4), n=32 |
| FAQ (informant rated)  (mean (sd), missing) | 17.6 (4.6), n=92 | | 20.4 (8.5), n=43 | | 22.4 (8.6), n=23 |
| Dependence (informant rated)  (mean (sd), missing) | 5.6 (2.6), n=80 | | 6.2 (2.9), n=42 | | 6.9 (3.0), n=32 |

*Note.* AD, Alzheimer’s disease; VaD, vascular dementia; FTD, frontotemporal dementia; PDD, Parkinson’s disease dementia; DLB, dementia with Lewy bodies; RSS, Relative Stress Scale; PAC, positive aspects of caregiving; RSE, Rosenberg Self-Esteen Scale; MMSE, mini-mental state examination; NPI, neuropsychiatric inventory; PAI, positive affect index; CESD_R, Center for Epidemiologic Studies Depression Scale-Revised; MSRS, modified social restriction scale; FAQ-I, functional activities questionnaire – informant-rated.

**Supplementary Table 9. Characteristics of caregivers and baseline scores on study variables for each class of stress**

| **Baseline measures** | **C1 High**  **(8.3%)** | **C2 Middle (46.1%)** | **C3 Low**  **(39.5%)** | **C4 Increasing**  **(6.1%)** |
| --- | --- | --- | --- | --- |
| Caregiver age (mean, sd) | 67.9 (11.6) | 68.9 (10.6) | 69.8 (11.0) | 70.0 (10.6) |
| Caregiver sex (%)  Male  Female | 16.4%  83.6% | 26.0%  74.0% | 39.4%  60.6% | 29.4%  70.6% |
| Diagnosis (%)  AD  VaD  Mixed AD/VaD  FTD  PDD/DLB  Other | 46.9%  10.9%  23.1%  4.9%  10.7%  3.5% | 55.1%  9.5%  19.8%  4.7%  8.6%  2.3% | 58.3%  11.0%  20.7%  3.7%  4.7%  1.4% | 60.8%  7.3%  24.8%  1.8%  4.7%  0.5% |
| Caregiver status (%)  Spouse/partner  Family/friend | 84.6%  15.4% | 84.7%  15.3% | 81.3%  18.7% | 82.5%  17.5% |
| Neuroticism (miniIPIP) (mean, sd) | 13.7 (3.1) | 11.5 (2.9) | 9.6 (2.9) | 9.8 (2.8) |
| Self-esteem (RSE) (mean, sd) | 28.2 (4.2) | 30.2 (4.3) | 32.7 (4.3) | 32.5 (4.3) |
| MMSE (mean, sd) | 22.6 (3.7) | 22.9 (3.6) | 23.5 (3.7) | 23.5 (3.7) |
| NPI distress (mean, sd) | 14.5 (8.3) | 7.6 (6.0) | 3.1 (3.7) | 2.8 (3.7) |
| NPI severity (mean, sd) | 10.9 (5.7) | 6.9 (4.7) | 3.5 (3.5) | 3.2 (3.4) |
| NPI symptoms (mean, sd) | 5.8 (2.4) | 4.2 (2.3) | 2.5 (2.1) | 2.3 (2.0) |
| Competence (mean, sd) | 7.9 (1.9) | 8.8 (1.5) | 9.7 (1.6) | 9.8 (1.7) |
| PAI current (mean, sd) | 18.9 (5.3) | 22.1 (4.6) | 25.1 (3.7) | 25.4 (3.8) |
| Self-rated health (mean, sd) | 3.7 (1.1) | 3.9 (1.1) | 4.4 (1.1) | 4.2 (1.1) |
| Coping (%)  Never/sometimes  Often/always | 53.1%  46.9% | 30.6%  69.4% | 13.6%  86.4% | 16.5%  83.5% |
| CESD-R (mean, sd) | 17.5 (11.9) | 8.5 (7.6) | 3.9 (4.9) | 4.1 (5.1) |
| MSRS (mean, sd) | 4.3 (1.3) | 3.7 (1.3) | 3.1 (1.3) | 3.3 (1.4) |
| PAC (mean, sd) | 24.9 (8.4) | 27.6 (7.0) | 29.7 (7.2) | 29.5 (8.2) |
| FAQ-I (mean, sd) | 23.1 (6.4) | 19.8 (7.5) | 14.2 (8.6) | 13.2 (8.9) |
| Dependence-I (mean, sd) | 7.4 (2.3) | 6.3 (2.3) | 4.5 (2.5) | 4.5 (2.4) |

*Note.* AD, Alzheimer’s disease; VaD, vascular dementia; FTD, frontotemporal dementia; PDD, Parkinson’s disease dementia; DLB, dementia with Lewy bodies; RSE, Rosenberg Self-Esteen Scale; MMSE, mini-mental state examination; NPI, neuropsychiatric inventory; PAI, positive affect index; CESD-R, Center for Epidemiologic Studies Depression Scale-Revised; MSRS, modified social restriction scale; RSS, PAC, positive aspects of caregiving; FAQ-I, functional activities questionnaire – informant-rated.

**Supplementary Table 10.** **Descriptive statistics of longitudinal measures across time points by stress class**

| **Measures** | **C1 High**  **(8.3%)** | **C2 Middle (46.1%)** | **C3 Low**  **(39.5%)** | **C4 Increasing**  **(6.1%)** |
| --- | --- | --- | --- | --- |
| MMSE (mean, sd) | T1: 22.6 (3.7)  T2: 20.8 (5.1)  T3: 21.0 (5.5) | T1: 22.9 (3.6)  T2: 20.9 (5.1)  T3: 19.5 (6.5) | T1: 23.5 (3.7)  T2: 22.5 (4.9)  T3: 21.6 (5.7) | T1: 23.5 (3.7)  T2: 21.3 (4.6)  T3: 19.4 (6.4) |
| NPI distress (mean, sd) | T1: 14.5 (8.3)  T2: 12.6 (8.1)  T3: 13.7 (10.3) | T1: 7.6 (6.0)  T2: 8.9 (6.8)  T3: 10.2 (7.3) | T1: 3.1 (3.7)  T2: 3.5 (4.4)  T3: 4.1 (4.5) | T1: 2.8 (3.7)  T2: 5.2 (5.0)  T3: 9.0 (6.4) |
| NPI severity (mean, sd) | T1: 10.9 (5.7)  T2: 9.9 (5.7)  T3: 10.0 (6.1) | T1: 6.9 (4.7)  T2: 7.8 (5.1)  T3: 8.6 (5.3) | T1: 3.5 (3.5)  T2: 3.7 (3.8)  T3: 4.3 (4.0) | T1: 3.2 (3.4)  T2: 5.3 (4.0)  T3: 8.3 (5.1) |
| NPI symptoms (mean, sd) | T1: 5.8 (2.4)  T2: 5.5 (2.6)  T3: 5.5 (2.5) | T1: 4.2 (2.3)  T2: 4.5 (2.4)  T3: 5.0 (2.3) | T1: 2.5 (2.1)  T2: 2.6 (2.2)  T3: 2.9 (2.3) | T1: 2.3 (2.0)  T2: 3.4 (2.1)  T3: 4.8 (2.3) |
| Competence (mean, sd) | T1: 7.9 (1.9)  T2: 8.1 (2.1)  T3: 8.3 (1.6) | T1: 8.8 (1.5)  T2: 8.7 (1.5)  T3: 8.6 (1.5) | T1: 9.7 (1.6)  T2: 9.5 (1.5)  T3: 9.5 (1.5) | T1: 9.8 (1.7)  T2: 9.4 (1.7)  T3: 8.8 (1.5) |
| PAI current (mean, sd) | T1: 18.9 (5.3)  T2: 18.6 (5.2)  T3: 18.1 (5.6) | T1: 22.1 (4.6)  T2: 21.2 (4.8)  T3: 20.5 (4.4) | T1: 25.1 (3.7)  T2: 24.3 (4.0)  T3: 24.1 (4.1) | T1: 25.4 (3.8)  T2: 23.3 (4.5)  T3: 22.1 (4.7) |
| Self-rated health (mean, sd) | T1: 3.7 (1.9)  T2: 3.2 (1.1)  T3: 3.3 (1.0) | T1: 3.9 (1.1)  T2: 3.7 (1.1)  T3: 3.7 (1.0) | T1: 4.4 (1.1)  T2: 4.3 (1.1)  T3: 4.2 (1.0) | T1: 4.2 (1.0)  T2: 4.0 (1.1)  T3: 3.6 (1.1) |
| Coping  % Never/sometimes | T1: 53.1%  T2: 63.3%  T3: 53.0% | T1: 30.6%  T2: 38.8%  T3: 44.8% | T1: 13.6%  T2: 20.2%  T3: 18.9% | T1: 16.5%  T2: 32.7%  T3: 39.1% |
| CESD-R (mean, sd) | T1: 17.5 (11.6)  T2: 18.0 (12.8)  T3: 19.5 (14.0) | T1: 8.5 (7.6)  T2: 10.5 (9.2)  T3: 11.7 (10.0) | T1: 3.9 (4.9)  T2: 4.5 (5.8)  T3: 4.6 (6.4) | T1: 4.1 (5.1)  T2: 6.9 (8.0)  T3: 8.6 (6.9) |
| MSRS (mean, sd) | T1: 4.3 (1.3)  T2: 4.4 (1.3)  T3: 4.4 (1.3) | T1: 3.7 (1.3)  T2: 4.0 (1.3)  T3: 4.2 (1.3) | T1: 3.1 (1.3)  T2: 3.3 (1.4)  T3: 3.4 (1.4) | T1: 3.3 (1.4)  T2: 3.7 (1.5)  T3: 4.2 (1.3) |
| PAC (mean, sd) | T1: 24.9 (8.4)  T2: 24.4 (8.7)  T3: 26.3 (8.6) | T1: 27.6 (7.0)  T2: 27.0 (7.4)  T3: 26.4 (7.5) | T1: 29.7 (7.2)  T2: 29.9 (7.1)  T3: 29.8 (7.6) | T1: 29.5 (8.2)  T2: 28.7 (8.5)  T3: 27.4 (8.3) |
| FAQ-I (mean, sd) | T1: 23.1 (6.4)  T2: 23.8 (7.2)  T3: 25.1 (7.4) | T1: 19.8 (7.5)  T2: 23.1 (7.2)  T3: 24.9 (7.0) | T1: 14.2 (8.6)  T2: 17.1 (8.9)  T3: 19.1 (9.3) | T1: 13.2 (8.9)  T2: 18.9 (8.8)  T3: 24.3 (8.0) |
| Dependence-I (mean, sd) | T1: 7.4 (2.3)  T2: 7.7 (2.5)  T3: 8.2 (2.9) | T1: 6.3 (2.3)  T2: 7.1 (2.7)  T3: 7.9 (2.7) | T1: 4.5 (2.5)  T2: 5.1 (2.7)  T3: 5.7 (2.9) | T1: 4.5 (2.4)  T2: 5.6 (2.9)  T3: 7.2 (2.6) |

*Note.* AD, Alzheimer’s disease; VaD, vascular dementia; FTD, frontotemporal dementia; PDD, Parkinson’s disease dementia; DLB, dementia with Lewy bodies; MMSE, mini-mental state examination; NPI, neuropsychiatric inventory; PAI, positive affect index; CESD-R, Center for Epidemiologic Studies Depression Scale-Revised; MSRS, modified social restriction scale; PAC, positive aspects of caregiving; FAQ-I, functional activities questionnaire – informant-rated.

**Table 11. Characteristics of caregivers and baseline scores on study variables for each class of PAC**

| **Baseline measures** | **C1 High**  **(15.2%)** | **C2 Middle**  **(67.6%)** | **C3 Low**  **(9.3%)** | **C4 Increasing**  **(3.4%)** | **C5 Decreasing**  **(4.5%)** |
| --- | --- | --- | --- | --- | --- |
| Age (mean, sd) | 69.6 (12.7) | 69.1 (10.9) | 69.0 (8.5) | 69.4 (8.3) | 69.1 (10.5) |
| Sex (%)  Male  Female | 41.2%  58.8% | 30.2%  69.8% | 20.5%  79.5% | 20.3%  79.7% | 26.2%  73.8% |
| Diagnosis (%)  AD  VaD  Mixed AD/VaD  FTD  PDD/DLB  Other | 57.1%  9.9%  21.2%  4.3%  6.1%  1.4% | 55.1%  10.9%  20.9%  3.7%  7.1%  2.3% | 57.4%  7.1%  19.8%  6.6%  7.3%  1.8% | 67.6%  8.0%  15.6%  4.2%  4.1%  0.8% | 52.4%  7.1%  25.0%  4.1%  9.8%  1.5% |
| Caregiver status (%)  Spouse/partner  Family/friend | 80.6%  19.4% | 82.0%  18.0% | 91.8%  8.2% | 84.0%  16.0% | 82.7%  17.3% |
| Neuroticism (miniIPIP) (mean, sd) | 10.4 (3.0) | 10.9 (3.1) | 11.5 (3.5) | 11.2 (3.2) | 10.8 (3.1) |
| RSE (mean, sd) | 32.3 (4.2) | 30.9 (4.4) | 30.8 (4.9) | 32.0 (4.8) | 30.4 (4.8) |
| MMSE (mean, sd) | 22.8 (3.5) | 23.1 (3.7) | 23.6 (3.6) | 23.0 (3.8) | 23.4 (3.6) |
| NPI distress (mean, sd) | 4.8 (5.9) | 6.1 (6.2) | 8.1 (7.4) | 7.0 (7.4) | 5.7 (5.7) |
| NPI severity (mean, sd) | 5.0 (4.8) | 5.7 (4.7) | 6.8 (5.3) | 5.7 (5.4) | 5.6 (4.8) |
| NPI symptoms (mean, sd) | 3.2 (2.6) | 3.5 (2.4) | 4.1 (2.6) | 3.4 (2.7) | 3..4 (2.5) |
| Competence (mean, sd) | 10.1 (1.5) | 9.1 (1.6) | 8.4 (1.8) | 8.7 (1.9) | 9.1 (1.6) |
| PAI current (mean, sd) | 25.2 (3.7) | 23.2 (4.5) | 20.5 (5.6) | 22.3 (6.0) | 22.5 (4.8) |
| Self-rated health (mean, sd) | 4.1 (1.0) | 4.1 (1.1) | 4.0 (1.2) | 4.2 (1.1) | 4.0 (1.2) |
| Coping (%)  Never/sometimes  Often/always | 8.5%  91.5% | 24.8%  75.2% | 43.5%  56.5% | 42.4%  57.6% | 32.8%  67.2% |
| CESD-R (mean, sd) | 5.4 (6.9) | 7.0 (7.7) | 9.8 (9.8) | 8.5 (10.6) | 8.2 (8.7) |
| MSRS (mean, sd) | 3.1 (1.3) | 3.5 (1.3) | 4.2 (1.4) | 3.8 (1.5) | 3.4 (1.4) |
| RSS (mean, sd) | 15.5 (9.2) | 18.7 (9.5) | 24.1 (10.5) | 22.0 (11.7) | 20.2 (9.4) |
| FAQ-I (mean, sd) | 17.5 (8.9) | 17.4 (8.6) | 18.2 (8.2) | 17.6 (8.9) | 17.7 (8.6) |
| Dependence-I (mean, sd) | 5.4 (2.8) | 5.6 (2.6) | 5.5 (2.3) | 5.4 (2.4) | 5.6 (2.4) |

*Note.* AD, Alzheimer’s disease; VaD, vascular dementia; FTD, frontotemporal dementia; PDD, Parkinson’s disease dementia; DLB, dementia with Lewy bodies; RSE, Rosenberg self-esteem scale; MMSE, mini-mental state examination; NPI, neuropsychiatric inventory; PAI, positive affect index; CESD-R, Center for Epidemiologic Studies Depression Scale-Revised; MSRS, modified social restriction scale; RSS, relative stress scale; FAQ-I, functional activities questionnaire – informant-rated.

**Supplementary Table 12. Descriptive statistics of longitudinal measures over time by PAC class**

| **Measures** | **C1 High**  **(15.2%)** | **C2 Middle**  **(67.6%)** | **C3 Low**  **(9.3%)** | **C4 Increasing**  **(3.4%)** | **C5 Decreasing**  **(4.5%)** |
| --- | --- | --- | --- | --- | --- |
| MMSE (mean, sd) | T1: 22.8 (3.5)  T2: 21.5 (4.9)  T3: 20.2 (5.9) | T1: 23.1 (3.7)  T2: 21.7 (5.1)  T3: 20.7 (6.2) | T1: 23.6 (3.6)  T2: 21.3 (4.9)  T3: 20.2 (6.1) | T1: 23.0 (3.8)  T2: 21.3 (5.2)  T3: 19.7 (6.4) | T1: 23.4 (3.6)  T2: 21.5 (5.1)  T3: 19.6 (6.8) |
| NPI distress (mean, sd) | T1: 4.8 (5.9)  T2: 5.1 (5.8)  T3: 5.8 (6.4) | T1: 6.1 (6.2)  T2:6.5 (6.5)  T3: 7.5 (7.3) | T1: 8.1 (7.4)  T2: 9.4 (7.0)  T3: 10.4 (6.9) | T1: 7.0 (7.4)  T2: 7.7 (6.7)  T3: 7.2 (6.0) | T1: 5.7 (5.7)  T2: 7.7 (7.8)  T3: 8.0 (7.5) |
| NPI severity (mean, sd) | T1: 5.0 (4.8)  T2: 5.2 (4.8)  T3: 6.0 (5.4) | T1: 5.7 (4.7)  T2: 5.9 (5.0)  T3: 6.6 (5.3) | T1: 6.8 (5.3)  T2: 8.0 (5.4)  T3: 8.6 (4.6) | T1: 5.7 (5.4)  T2: 6.5 (5.3)  T3: 6.5 (4.0) | T1: 5.6 (4.8)  T2: 6.9 (6.0)  T3: 7.2 (6.0) |
| NPI symptoms (mean, sd) | T1: 3.2 (2.6)  T2: 3.3 (2.6)  T3: 3.7 (2.7) | T1: 3.5 (2.4)  T2: 3.7 (2.5)  T3: 4.1 (2.6) | T1: 4.1 (2.6)  T2: 4.7 (2.6)  T3: 4.9 (2.2) | T1: 3.4 (2.7)  T2: 4.0 (2.8)  T3: 4.0 (2.0) | T1: 3.4 (2.4)  T2: 3.8 (2.6)  T3: 4.0 (2.6) |
| Competence (mean, sd) | T1: 10.1 (1.5)  T2: 9.9 (1.5)  T3: 9.7 (1.4) | T1: 9.1 (1.6)  T2: 9.0 (1.5)  T3: 9.0 (1.5) | T1: 8.4 (1.8)  T2: 8.3 (1.8)  T3: 8.2 (1.7) | T1: 8.7 (1.9)  T2: 8.7 (1.8)  T3: 9.0 (1.6) | T1: 9.1 (1.6)  T2: 8.7 (1.7)  T3: 8.2 (1.8) |
| PAI current (mean, sd) | T1: 25.2 (3.7)  T2: 24.1 (4.2)  T3: 23.9 (4.2) | T1: 23.2 (4.5)  T2: 22.5 (4.7)  T3: 22.1 (4.6) | T1: 20.5 (5.6)  T2: 19.7 (5.2)  T3: 19.6 (5.2) | T1: 22.3 (6.0)  T2: 21.3 (4.8)  T3: 22.3 (4.9) | T1: 22.5 (4.8)  T2: 21.4 (5.3)  T3: 20.0 (5.0) |
| Self-rated health (mean, sd) | T1: 4.1 (1.0)  T2: 4.1 (1.1)  T3: 4.0 (1.0) | T1: 4.1 (1.1)  T2: 3.9 (1.1)  T3: 3.9 (1.1) | T1: 4.0 (1.2)  T2: 3.9 (1.2)  T3:3.9 (1.0) | T1: 4.2 (1.1)  T2: 4.1 (1.2)  T3: 3.8 (1.1) | T1: 4.0 (1.2)  T2: 3.8 (1.2)  T3: 3.6 (1.1) |
| Coping  % Never/sometimes | T1: 8.5%  T2: 13.9%  T3: 12.0% | T1: 24.8%  T2: 32.1%  T3: 32.7% | T1: 43.5%  T2: 55.9%  T3: 55.5% | T1: 42.4%  T2: 41.2%  T3: 40.7% | T1: 32.8%  T2: 43.1%  T3: 60.9% |
| CESD-R (mean, sd) | T1: 5.4 (6.9)  T2: 6.5 (8.9)  T3: 6.9 (9.3) | T1: 7.0 (7.7)  T2: 8.2 (9.1)  T3: 8.9 (9.9) | T1: 9.8 (9.8)  T2: 10.9 (8.6)  T3: 9.7 (7.2) | T1: 8.5 (10.6)  T2: 9.6 (8.6)  T3: 7.8 (7.1) | T1: 8.2 (8.7)  T2: 10.2 (10.1)  T3: 13.5 (12.0) |
| MSRS (mean, sd) | T1: 3.1 (1.3)  T2: 3.4 (1.4)  T3: 3.4 (1.3) | T1: 3.5 (1.3)  T2: 3.7 (1.4)  T3: 3.8 (1.4) | T1: 4.2 (1.4)  T2: 4.5 (1.3)  T3: 4.6 (1.3) | T1: 3.8 (1.5)  T2: 3.8 (1.5)  T3: 4.1 (1.5) | T1: 3.4 (1.4)  T2: 3.6 (1.3)  T3: 3.9 (1.5) |
| RSS (mean, sd) | T1: 15.5 (9.2)  T2: 18.5 (10.2)  T3: 20.1 (10.9) | T1: 18.7 (9.5)  T2: 20.9 (9.7)  T3: 22.7 (10.1) | T1: 24.1 (10.5)  T2: 27.9 (9.5)  T3: 28.3 (8.6) | T1: 22.0 (11.7)  T2: 25.2 (10.4)  T3: 24.4 (9.5) | T1: 20.2 (9.4)  T2: 24.2 (9.6)  T3: 27.8 (9.9) |
| FAQ-I (mean, sd) | T1: 17.5 (8.9)  T2: 19.7 (8.3)  T3: 22.3 (8.6) | T1: 17.4 (8.6)  T2: 20.3 (8.7)  T3: 22.1 (8.8) | T1: 18.2 (8.2)  T2: 22.0 (7.7)  T3: 23.4 (7.6) | T1: 17.6 (8.9)  T2: 20.7 (8.8)  T3: 22.3 (8.1) | T1: 17.7 (8.6)  T2: 21.4 (8.5)  T3: 24.2 (8.2) |
| Dependence-I (mean, sd) | T1: 5.4 (2.8)  T2: 6.2 (3.0)  T3: 7.0 (2.9) | T1: 5.6 (2.6)  T2: 6.2 (2.9)  T3: 6.9 (3.1) | T1: 5.5 (2.3)  T2: 6.4 (2.5)  T3: 7.0 (2.7) | T1: 5.4 (2.4)  T2: 6.5 (3.0)  T3: 6.7 (2.8) | T1: 5.6 (2.4)  T2: 6.5 (2.9)  T3: 7.3 (3.0) |

*Note.* AD, Alzheimer’s disease; VaD, vascular dementia; FTD, frontotemporal dementia; PDD, Parkinson’s disease dementia; DLB, dementia with Lewy bodies; MMSE, mini-mental state examination; NPI, neuropsychiatric inventory; PAI, positive affect index; CESD-R, Center for Epidemiologic Studies Depression Scale-Revised; MSRS, modified social restriction scale; RSS, relative stress scale; FAQ-I, functional activities questionnaire – informant-rated. Models are adjusted for caregiver sex, caregiver age, diagnosis type, caregiver status.

**Supplementary Table 13. Characteristics of baseline scores on study variables for each class of joint stress/PAC**

| **Baseline measures** | **C1**  **(72.2%)** | **C2**  **(15.2%)** | **C3**  **(12.5%)** |
| --- | --- | --- | --- |
| Caregiver age (mean, sd) | 69.1 (10.9) | 69.8 (12.5) | 69.2 (8.4) |
| Caregiver sex (%)  Male  Female | 30.2%  69.8% | 41.2%  58.8% | 19.9%  80.1% |
| Diagnosis (%)  AD  VaD  Mixed AD/VaD  FTD  PDD/DLB  Other | 55.4%  11.1%  20.8%  3.4%  3.7%  2.7% | 58.0%  9.7%  22.3%  3.3%  4.4%  2.2% | 61.2%  9.0%  17.4%  5.3%  6.1%  0.9% |
| Caregiver status (%)  Spouse/partner  Family/friend | 82.1%  17.9% | 80.9%  19.1% | 89.8%  10.2% |
| Neuroticism (miniIPIP) (mean, sd) | 10.9 (3.1) | 10.4 (3.0) | 11.5 (3.4) |
| RSE (mean, sd) | 30.9 (4.4) | 32.4 (4.2) | 31.0 (4.8) |
| MMSE (mean, sd) | 23.2 (3.7) | 22.8 (3.5) | 23.4 (3.7) |
| NPI distress (mean, sd) | 6.0 (6.1) | 4.7 (5.9) | 8.0 (7.5) |
| NPI severity (mean, sd) | 5.7 (4.7) | 5.0 (4.7) | 6.7 (5.4) |
| NPI symptoms (mean, sd) | 3.5 (2.4) | 3.2 (2.5) | 4.0 (2.7) |
| Competence (mean, sd) | 9.1 (1.6) | 10.1 (1.5) | 8.4 (1.8) |
| PAI current (mean, sd) | 23.2 (4.5) | 25.2 (3.7) | 20.9 (5.7) |
| Self-rated health (mean, sd) | 4.1 (1.1) | 4.1 (1.0) | 4.0 (1.1) |
| Coping (%)  Never/sometimes  Often/always | 25.0%  75.0% | 8.6%  91.4% | 43.9%  56.1% |
| CESD-R (mean, sd) | 7.1 (7.7) | 5.3 (6.7) | 9.6 (10.1) |
| MSRS (mean, sd) | 3.5 (1.3) | 3.1 (1.3) | 4.1 (1.4) |
| FAQ-I (mean, sd) | 17.5 (8.6) | 17.3 (9.0) | 18.2 (8.3) |
| Dependence-I (mean, sd) | 5.6 (2.6) | 5.4 (2.8) | 5.5 (2.3) |

*Note.* AD, Alzheimer’s disease; VaD, vascular dementia; FTD, frontotemporal dementia; PDD, Parkinson’s disease dementia; DLB, dementia with Lewy bodies; RSE, Rosenberg self-esteem scale; MMSE, mini-mental state examination; NPI, neuropsychiatric inventory; PAI, positive affect index; CESD-R, Center for Epidemiologic Studies Depression Scale-Revised; MSRS, modified social restriction scale; FAQ-I, functional activities questionnaire – informant-rated.

**Supplementary Table 14.** **Associations of baseline measures with classes of joint**

**stress/PAC**

| **Baseline measures** | **C2 (15.2%)**  **(ref: C1)**  OR (95% CI) | **C3 (12.5%)**  **(ref: C1)**  OR (95% CI) |
| --- | --- | --- |
| Caregiver age | 1.01 (0.98 – 1.03) | 0.98 (0.96 – 1.01) |
| Caregiver sex (male) | 1.89 (1.23 – 2.92)* | 0.50 (0.29 – 0.86)* |
| Diagnosis  AD  VaD  Mixed AD/VaD  FTD  PDD/DLB  Other | Ref  0.80 (0.38 – 1.67)  1.00 (0.60 – 1.68)  1.21 (0.45 – 3.26)  1.00 (0.44 – 2.27)  0.75 (0.15 – 3.78) | Ref  0.50 (0.21 – 1.17)  0.80 (0.46 – 1.39)  1.30 (0.52 – 3.25)  0.67 (0.29 – 1.56)  0.52 (0.10 – 2.76) |
| Caregiver status (family/friend) | 1.40 (0.76 – 2.57) | 0.24 (0.10 – 0.73)* |
| Neuroticism (miniIPIP) | 0.95 (0.89 – 1.01) | 1.04 (0.96 – 1.12) |
| RSE | 1.10 (1.06 – 1.15)* | 1.03 (0.97 – 1.08) |
| MMSE | 0.99 (0.93 – 1.04) | 0.99 (0.94 – 1.05) |
| NPI distress | 0.95 (0.91 – 1.00) | 1.05 (1.01 – 1.08)* |
| NPI severity | 0.96 (0.91 - 1.01) | 1.04 (1.00 – 1.09)* |
| NPI symptoms | 0.93 (0.85 – 1.03) | 1.07 (0.98 – 1.17) |
| Competence | 1.65 (1.43 – 1.90)* | 0.72 (0.62 – 0.85)* |
| PAI current | 1.16 (1.10 – 1.23)* | 0.90 (0.86 – 0.94)* |
| Self-rated health | 0.97 (0.83 – 1.24) | 0.99 (0.77 – 1.25) |
| Coping (never/sometimes vs often/always) | 3.07 (2.17 – 4.36)* | 0.54 (0.40 – 0.74)* |
| CESD | 0.94 (0.90 – 0.99)* | 1.03 (1.01 – 1.06)* |
| MSRS | 0.78 (0.65 – 0.93)* | 1.40 (1.19 – 1.64)* |
| FAQ-I | 1.00 (0.97 – 1.02) | 1.02 (1.00 – 1.05)* |
| Dependence-I | 0.97 (0.89 – 1.06) | 1.00 (0.93 – 1.08) |

*Note.* OR, odds ratio; CI, confidence intervals; AD, Alzheimer’s disease; VaD, vascular dementia; FTD, frontotemporal dementia; PDD, Parkinson’s disease dementia; DLB, dementia with Lewy bodies; RSE, Rosenberg self-esteem scale; MMSE, mini-mental state examination; NPI, neuropsychiatric inventory; PAI, positive affect index; CESD-R, Center for Epidemiologic Studies Depression Scale-Revsied; MSRS, modified social restriction scale; FAQ-I, functional activities questionnaire – informant-rated. Models were adjusted for caregiver sex, caregiver age, diagnosis type, caregiver status.

**Supplementary Table 15. Descriptive statistics of longitudinal measures across time points by joint stress/PAC classes**

| **Measures** | **C1**  **(72.2%)** | **C2**  **(15.2%)** | **C3**  **(12.5%)** |
| --- | --- | --- | --- |
| MMSE (mean, sd) | T1: 23.2 (3.7)  T2: 21.6 (5.1)  T3: 20.6 (6.2) | T1: 22.8 (3.5)  T2: 21.5 (4.9)  T3: 20.1 (5.9) | T1: 23.4 (3.7)  T2: 21.3 (5.0)  T3: 20.2 (6.2) |
| NPI distress (mean, sd) | T1: 6.0 (6.1)  T2: 6.5 (6.6)  T3: 7.5 (7.2) | T1: 4.7 (5.9)  T2: 5.0 (5.8)  T3: 5.8 (6.5) | T1: 8.0 (7.5)  T2: 9.0 (7.0)  T3: 9.8 (6.8) |
| NPI severity (mean, sd) | T1: 5.7 (4.7)  T2: 6.0 (5.0)  T3: 6.6 (5.3) | T1: 5.0 (4.7)  T2: 5.1 (4.8)  T3: 6.1 (5.4) | T1: 6.7 (5.4)  T2: 7.7 (5.4)  T3: 8.2 (4.5) |
| NPI symptoms (mean, sd) | T1: 3.5 (2.4)  T2: 3.6 (2.5)  T3: 4.1 (2.6) | T1: 3.2 (2.5)  T2: 3.3 (2.6)  T3: 3.8 (2.7) | T1: 4.0 (2.7)  T2: 4.5 (2.6)  T3: 4.8 (2.1) |
| Competence (mean, sd) | T1: 9.1 (1.6)  T2: 9.0 (1.5)  T3: 8.9 (1.5) | T1: 10.1 (1.5)  T2: 9.9 (1.5)  T3: 9.7 (1.5) | T1: 8.4 (1.8)  T2: 8.4 (1.8)  T3: 8.3 (1.7) |
| PAI current (mean, sd) | T1: 23.2 (4.5)  T2: 22.5 (4.7)  T3: 22.1 (4.7) | T1: 25.2 (3.7)  T2: 24.2 (4.2)  T3: 23.9 (4.3) | T1: 20.9 (5.7)  T2: 20.1 (5.1)  T3: 20.2 (5.2) |
| Self-rated health (mean, sd) | T1: 4.1 (1.1)  T2: 3.9 (1.1)  T3: 3.9 (1.1) | T1: 4.1 (1.0)  T2: 4.1 (1.1)  T3: 4.0 (1.0) | T1: 4.0 (1.1)  T2: 3.9 (1.2)  T3: 3.9 (1.0) |
| Coping  % Never/sometimes | T1: 25.0%  T2: 32.3%  T3: 34.1% | T1: 8.6%  T2: 14.8%  T3: 12.5% | T1: 43.9%  T2: 53.7%  T3: 53.2% |
| CESD-R (mean, sd) | T1: 7.1 (7.7)  T2: 8.3 (9.2)  T3: 9.1 (10.1) | T1: 5.3 (6.7)  T2: 6.3 (8.8)  T3: 6.8 (9.1) | T1: 9.6 (10.1)  T2: 10.6 (8.5)  T3: 9.3 (7.2) |
| MSRS (mean, sd) | T1: 3.5 (1.3)  T2: 3.7 (1.4)  T3: 3.8 (1.4) | T1: 3.1 (1.3)  T2: 3.4 (1.4)  T3: 3.4 (1.4) | T1: 4.1 (1.4)  T2: 4.3 (1.4)  T3: 4.5 (1.3) |
| FAQ-I (mean, sd) | T1: 17.5 (8.6)  T2: 20.3 (8.6)  T3: 22.2 (8.8) | T1: 17.3 (9.0)  T2: 19.5 (8.3)  T3: 22.3 (8.5) | T1: 18.2 (8.3)  T2: 21.8 (7.9)  T3: 23.3 (7.6) |
| Dependence-I (mean, sd) | T1: 5.6 (2.6)  T2: 6.2 (2.9)  T3: 6.9 (3.1) | T1: 5.4 (2.8)  T2: 6.1 (3.0)  T3: 7.0 (2.9) | T1: 5.5 (2.3)  T2: 6.5 (2.7)  T3: 7.0 (2.7) |

*Note.* MMSE, mini-mental state examination; NPI, neuropsychiatric inventory; PAI, positive affect index; CESD, Center for Epidemiologic Studies Depression Scale-Revised; MSRS, modified social restriction scale; FAQ-I, functional activities questionnaire – informant-rated.

**Supplementary Table 16. Associations of joint stress/PAC classes with measures across time points**

| **Baseline measures** | **C2 (15.2%)**  **(ref: C1)** | **C3 (12.5%)**  **(ref: C1)** |
| --- | --- | --- |
|  | RR (95% CI) | RR (95% CI) |
| MMSE | 1.04 (0.97 – 1.09) | 0.97 (0.92 – 1.03) |
| NPI distress | 0.97 (0.82 – 1.14) | 1.10 (0.94 – 1.28) |
| NPI severity | 1.02 (0.89 – 1.17) | 1.15 (1.00 – 1.31)* |
| NPI symptoms | 1.01 (0.92 – 1.12) | 1.05 (0.97 – 1.14) |
| Competence | 0.99 (0.96 – 1.02) | 1.00 (0.98 – 1.03) |
| PAI current | 1.00 (0.97 – 1.04) | 0.99 (0.96 – 1.02) |
| Self-rated health | 1.03 (0.98 – 1.07) | 1.02 (0.98 – 1.07) |
| CESD-R | 1.05 (0.91 – 1.21) | 1.03 (0.89 – 1.19) |
| MSRS | 1.00 (0.95 – 1.05) | 1.00 (0.96 – 1.06) |
| FAQ-I | 1.02 (0.94 – 1.10) | 1.02 (0.94 – 1.10) |
| Dependence-I | 1.05 (0.97 – 1.13) | 1.04 (0.96 – 1.12) |
|  | OR (95% CI) | OR (95% CI) |
| Coping | 0.67 (0.32 – 1.40) | 1.29 (0.74 – 2.25) |

*Note.* RR, rate ratio; OR, odds ratio; CI, confidence intervals; MMSE, mini-mental state examination; NPI, neuropsychiatric inventory; PAI, positive affect index; CESD, Center for Epidemiologic Studies Depression Scale-Revised; MSRS, modified social restriction scale; FAQ-I, functional activities questionnaire – informant-rated. Models are adjusted for caregiver sex, caregiver age, diagnosis type, caregiver status.

Models were adjusted for caregiver sex, caregiver age, diagnosis type, caregiver status.

**Supplementary Figure 1. Plots of GMM-CI and GMM-CV models for stress shown in Supplementary Table 1**


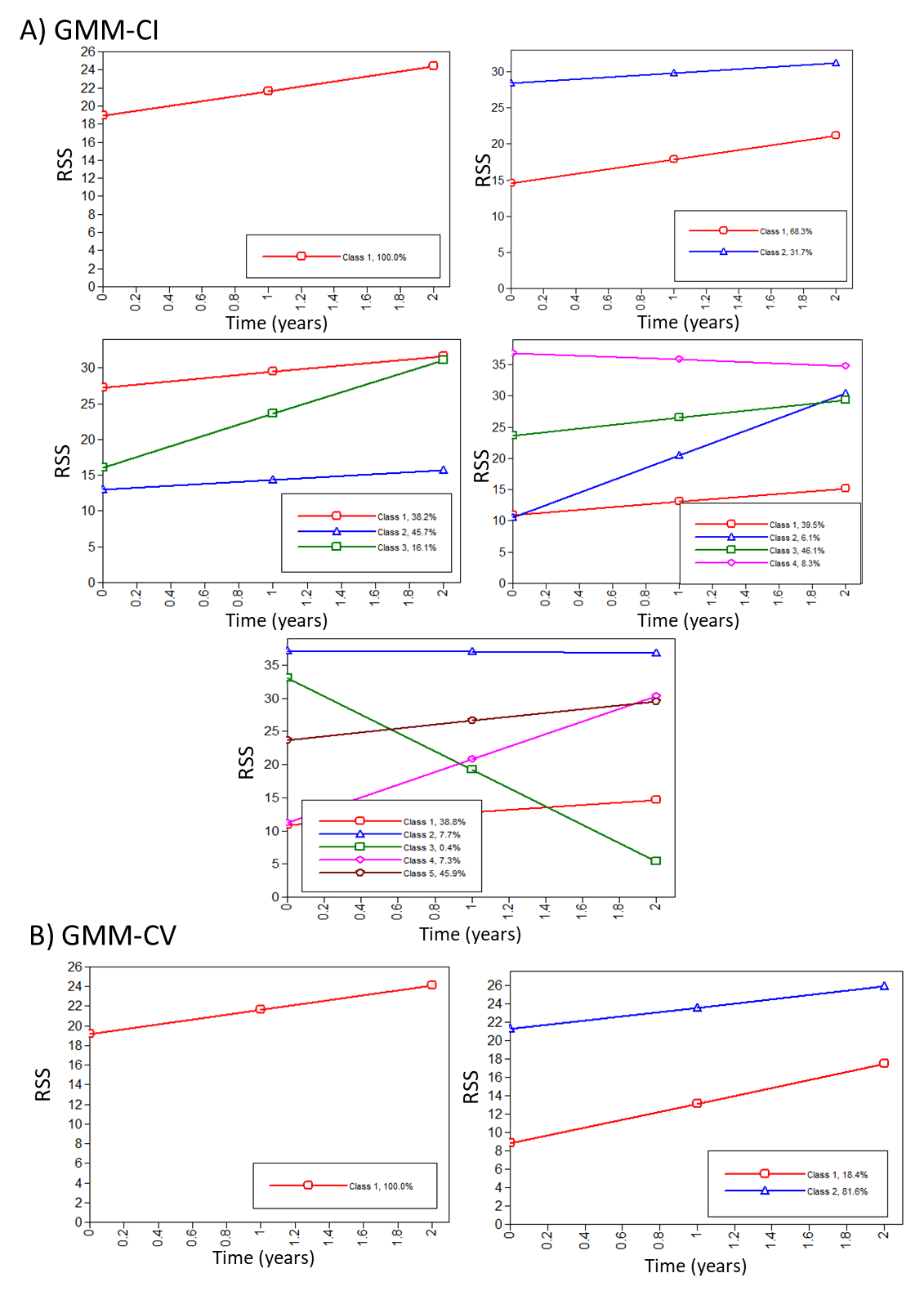


**Supplementary Figure 2. Plots of GMM-CI and GMM-CV models for positive aspects of caregiving shown in Supplementary Table 3**


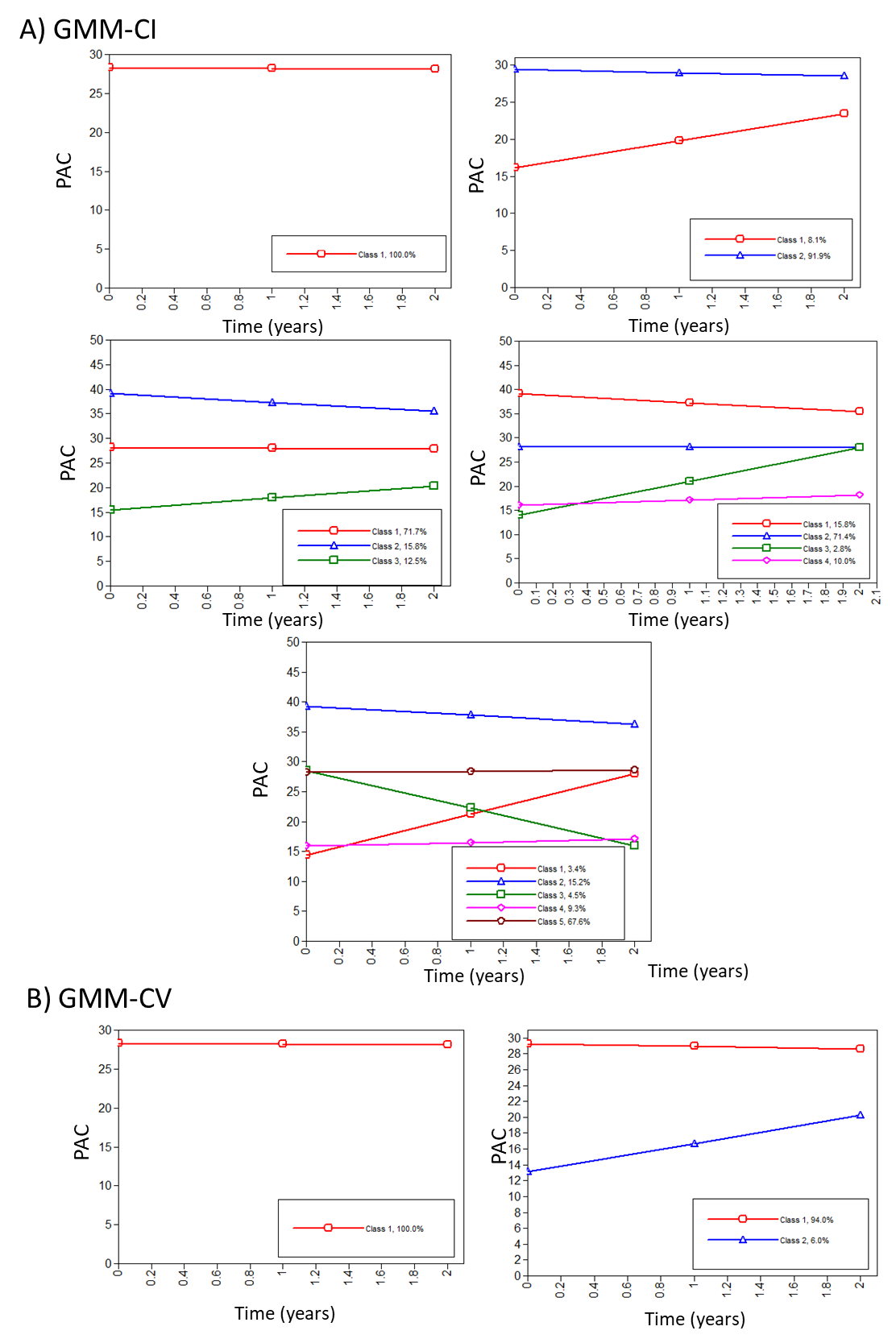


**Supplementary Figure 3.** **Plots of individual caregivers within the four classes of trajectories of stress shown in Figure 1B. The most likely class is allocated based on posterior probabilities for graphing purposes**


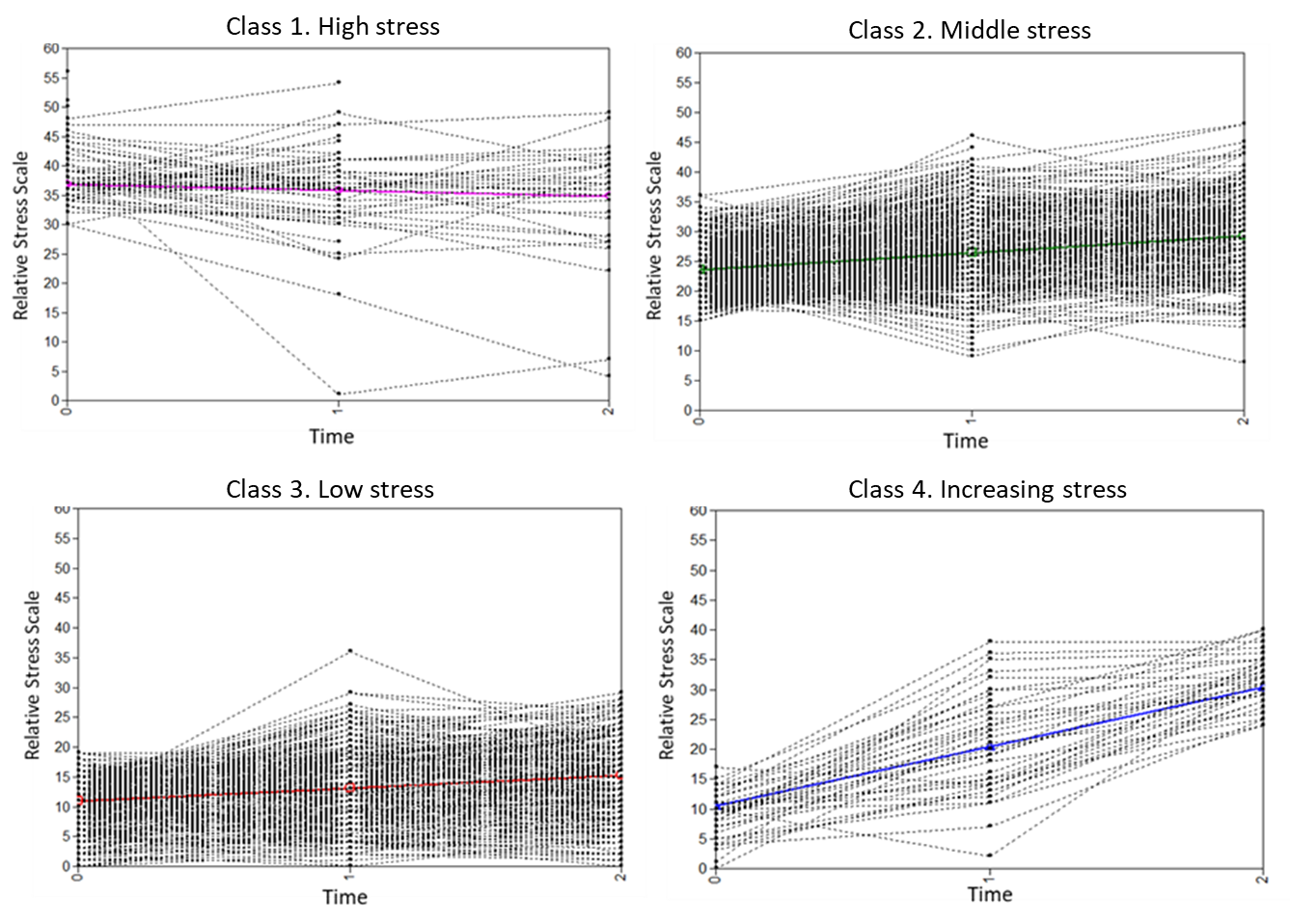


**Supplementary Figure 4. Plots of individual caregivers within the five classes of trajectories of PAC shown in Figure 2B. The most likely class is allocated based on posterior probabilities for graphing purposes**


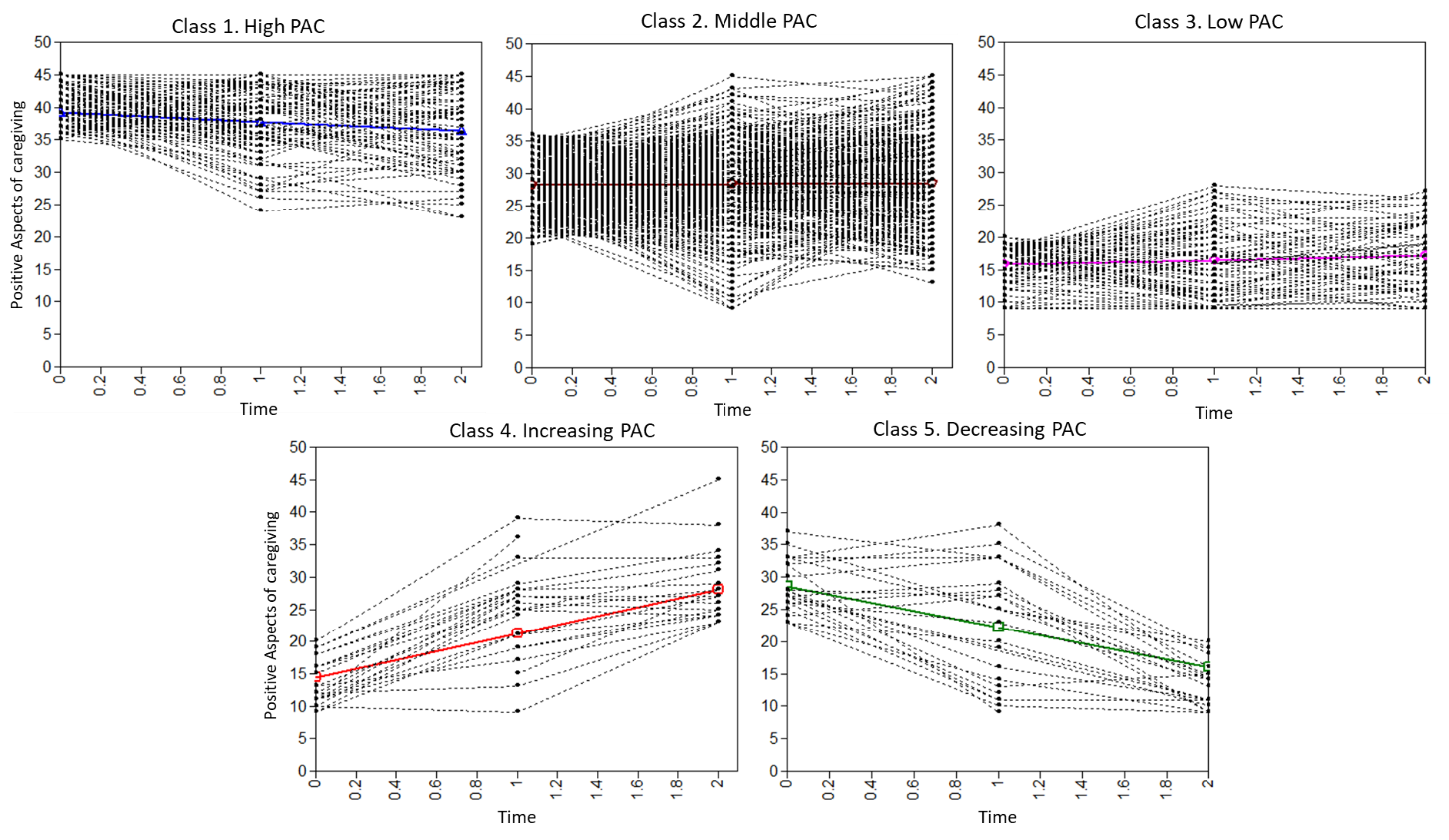


**Supplementary Figure 5. Extracted 3-class solution of joint stress/PAC growth mixture model**


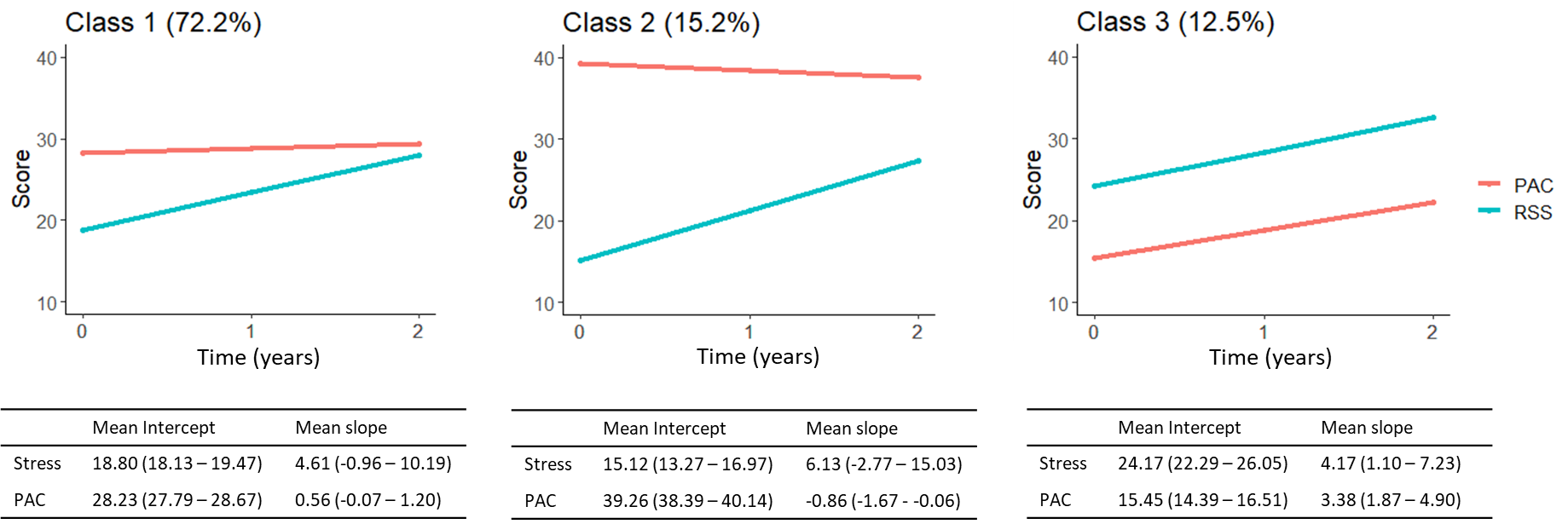

Supplement: gbae097_suppl_Supplementary_Materials [file gbae097_suppl_supplementary_materials.docx]
